# Zinc(ii) and cadmium(ii) amorphous metal–organic frameworks (aMOFs): study of activation process and high-pressure adsorption of greenhouse gases†

**DOI:** 10.1039/d1ra02938j

**Published:** 2021-06-04

**Authors:** Miroslav Almáši, Nikolas Király, Vladimír Zeleňák, Mária Vilková, Sandrine Bourrelly

**Affiliations:** Department of Inorganic Chemistry, Faculty of Science, P. J. Šafárik University Moyzesova 11 SK-041 54 Košice Slovak Republic miroslav.almasi@upjs.sk; NMR Laboratory, Faculty of Science, P. J. Šafárik University Moyzesova 11 SK-041 01 Košice Slovak Republic; Aix-Marseille University, CNRS, MADIREL Marseille Cedex 20 F-133 97 France

## Abstract

Two novel amorphous metal–organic frameworks (aMOFs) with chemical composition {[Zn_2_(MTA)]·4H_2_O·3DMF}_*n*_ (UPJS-13) and {[Cd_2_(MTA)]·5H_2_O·4DMF}_*n*_ (UPJS-14) built from Zn(ii) and Cd(ii) ions and extended tetrahedral tetraazo-tetracarboxylic acid (H_4_MTA) as a linker were prepared and characterised. Nitrogen adsorption measurements were performed on as-synthesized (AS), ethanol exchanged (EX) and freeze-dried (FD) materials at different activation temperatures of 60, 80, 100, 120, 150 and 200 °C to obtain the best textural properties. The largest surface areas of 830 m^2^ g^−1^ for UPJS-13 (FD) and 1057 m^2^ g^−1^ for UPJS-14 (FD) were calculated from the nitrogen adsorption isotherms for freeze-dried materials activated at mild activation temperature (80 °C). Subsequently, the prepared compounds were tested as adsorbents of greenhouse gases, carbon dioxide and methane, measured at high pressures. The maximal adsorption capacities were 30.01 wt% CO_2_ and 4.84 wt% CH_4_ for UPJS-13 (FD) and 24.56 wt% CO_2_ and 6.38 wt% CH_4_ for UPJS-14 (FD) at 20 bar and 30 °C.

## Abstract 2

Two novel amorphous metal–organic frameworks UPJS-13 and UPJS-14, constructed of Zn(ii)/Cd(ii) ions and extended tetrahedral linker were prepared, characterised and applied as adsorbents for carbon dioxide and methane.

## Graphical abstract

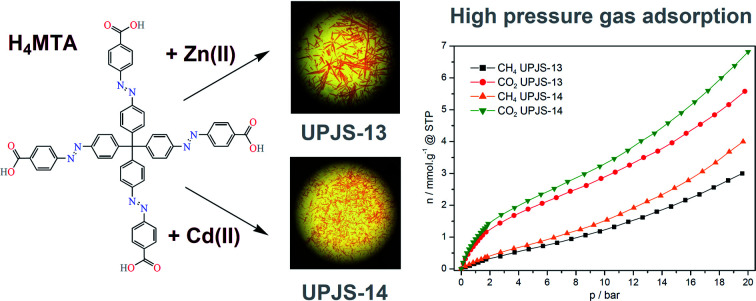

## 1 Introduction

Metal–organic frameworks (MOFs) are one of the most exciting classes of porous materials discovered in the past few decades. MOFs are crystalline materials constructed from organic molecules (linkers) coordinated to metal ions or clusters to form interesting polymeric frameworks with a larger surface area and the advantage of pore size changing. Due to the porosity and tunable textural properties, MOF materials find applications in gas storage and separation,^1,2^ heterogeneous catalysis,^3,4^ drug delivery,^5,6^ magnetic refrigeration,^7,8^ sensing^9,10^ and energy storage.^11,12^ A small but growing number of noncrystalline MOFs are steadily capturing scientific interest, though it is only recently that well-characterised examples of amorphous metal–organic frameworks (aMOFs) have started to appear.^13–15^ aMOFs retain the basic building blocks and connectivity of their crystalline counterparts, though they lack any long-range periodic order. aMOFs combine properties that are usually found in crystalline with the unique properties of the amorphous domain such as isotropy, absence of grain boundaries, abundant defects and active sites, and flexibility. Most aMOFs have been prepared by applying stress to a crystalline framework. To date, both static and hydrostatic pressures, heating, mechanical stress (ball milling, grinding), radiation, and also chemical treatment have been applied to crystalline MOFs to induce their collapse and amorphization.^16^ However, examples of aMOF materials which have been prepared in the amorphous state by direct synthesis are also known. It should be noted that only a minority number of compounds prepared by the mentioned way are known in the literature: a-UiO-66-SO_3_H (*S*_BET_ = 11 m^2^ g^−1^),^17^ a-FMM-120 (*S*_BET_ = 43 m^2^ g^−1^),^18^ CPPs-*X* (*X* = 1, 3, 5; *S*_BET_ = 522–588 m^2^ g^−1^),^19^ aZr-MOF(*X*) (*X* = 23–26; *S*_BET_ = 410–955 m^2^ g^−1^),^20^ NEU-*X* (*X* = 2–8; *S*_BET_ = 10–294 m^2^ g^−1^).^21–23^ As was described above, aMOFs have been mostly prepared in a top-down approach by applying stress and introducing disorder into the parent frameworks. In our work we developed bottom-up synthesis of aMOFs, with a high and permanent porosity. Amorphous MOFs offer many exciting opportunities for practical application, either as novel functional materials themselves or facilitating other processes, though the domain is mostly unexplored.^24^ Published articles dealing with aMOFs have been demonstrated to be of potential in several fields, *e.g.* catalysis,^25–28^ drug delivery,^29–33^ supercapacitors^34–36^ and irreversible harmful substance capture.^37,38^

To study the porosity and textural properties of porous materials is necessary to activate them and thus obtain an open structure. A typical activation process is a thermal activation, in which the process of releasing solvent molecules is caused by heat. This activation way often leads to the shrink or collapse of the polymeric frameworks causing a decrease of material's porosity or non-porosity. To reduce the activation temperature, a solvent exchange process is also used as a tool, in which high-boiling solvents are exchanged for low-boiling solvents and the thermal activation of porous material occurs at a lower temperature. The freeze-drying process, also known as lyophilisation or cryodesiccation, is a dehydration/desolvation process performed at low temperature, which involves freezing the material with guest molecules and subsequent sublimating the guests under low temperature and low pressure. Lyophilisation is a commercial process preliminary used in biological, biomedical processing, fine food production and preservation. The freeze-drying process has also found an application in the activation of porous materials.^39^ Large specific surface area and better textural properties can be obtained by exchanging of high boiling point solvents (*N*,*N*′-dimethylformamide (153 °C), *N*,*N*′-diethylformamide (176 °C), *N*,*N*′-dimethylacetamide (165 °C), dimethylsulfoxide (189 °C) *etc.*), that are typically located in the pores of as-synthesized material by the solvents with high melting point and high vapour pressure (benzene (5.5 °C, 75 mmHg at 20 °C), cyclohexane (4 °C, 77 mmHg at 20 °C), *tert*-butyl alcohol (23 °C, 31 mmHg at 20 °C), *o*-xylene (−23 °C, 7 mmHg at 20 °C) and compressed CO_2_) followed by evacuation at low temperature. For the organic nature of porous coordination polymers, many MOFs exhibit surface areas that are only a small fraction of predicted/calculated surfaces based on their crystal structures. It was shown that a freeze-drying process could achieve a higher surface area and the desired features of porous materials. For example, freeze-dried compounds [Cu_2_(MTB)(H_2_O)_2_]·6DEF·2H_2_O and [Cu_2_(MTC)(H_2_O)_2_] ·14DMF·5H_2_O, which also contain tetrahedral linkers (MTB = methanetetrabenzoate, MTC = methanetetra(biphenyl-*p*-carboxylate)) exhibited surface areas of 526 m^2^ g^−1^ and 791 m^2^ g^−1^ for evacuated materials (activation temperature 60 °C) and 1560 m^2^ g^−1^, 1020 m^2^ g^−1^ for freeze-dried samples, respectively.^40^ As is obvious from described values, lyophilisation is an efficient process for activating porous materials that can increase the surface area three times compared to standard thermal activation.

Global temperature has been rising since the first industrial revolution in the early 19th century, due to the emissions of large quantities of greenhouse gases. The main greenhouse gases are carbon dioxide and methane, but also nitrogen oxides and water vapour. Carbon dioxide is a major by-product of fossil fuel combustion, electricity generation and other anthropogenic activities. Major advances in carbon capture technology could provide power plants and road transport with an efficient and inexpensive way to remove carbon dioxide from their flue gas emissions, which is necessary to reduce greenhouse gas emissions to slow global warming and climate change. The 2030 Climate and Energy Framework commits all EU member states to a 40% reduction in greenhouse gas emissions over the coming years.^41^ For this reason, it is necessary to develop new and effective carbon dioxide and methane adsorbents as the main greenhouse gases. Many porous materials such as carbon dioxide adsorbents, *e.g.* MOFs or porous oxides, are modified with nitrogen-containing amine molecules to capture the CO_2_ (chemisorption) at low-temperature steam to flush out the CO_2_ for other uses or to sequester it underground are currently being developed and intensively studied. Examples are tetraamine-appended MOF Mg_2_(dobpdc) (dobpdc = 4,4′-dioxidobiphenyl-3,3′-dicarboxylate, *S*_BET_ = 2880 m^2^ g^−1^), which can store up 90 wt% CO_2_ (ref. 42) and amine grafted silica materials.^43–45^ In order to avoid CO_2_-intensive energy processes, CO_2_ molecules must be captured in porous materials by physisorption as a chemisorption process. However, physisorption brings new challenges, because there is a lack of selectivity for CO_2_ because no chemical bond is formed or when the functional groups are tuned to increase selectivity, the absorption of CO_2_ is limited.

In the present study, extended tetrahedral nitrogen-rich tetraazo-tetracarboxylic acid (H_4_MTA) was prepared by seven-step organic synthesis and used as a linker in the preparation of two novel aMOFs containing Zn(ii) (UPJS-13) and Cd(ii) (UPJS-14) ions. After characterisation, the textural properties of prepared materials were studied by nitrogen adsorption at −196 °C on as-synthesized (AS), ethanol exchanged (EX) and freeze-dried (FD) samples. The specific surface area (*S*_BET_) depended on the sample pretreatment (AS < EX < FD) and the activation temperature (60–200 °C). The highest *S*_BET_ values were obtained for FD materials after activation process at 80 °C: UPJS-13 (EX) 830 m^2^ g^−1^ and UPJS-14 (EX) 1057 m^2^ g^−1^. After finding the best activation conditions, the materials were tested as adsorbents of greenhouse gases in high-pressure adsorption measurements of carbon dioxide and methane up to 20 bar.

## 2 Experimental

### 2.1 Synthetic part

#### 2.1.1 Used chemicals

All chemicals used in the synthesis of methanetetrayltetrakis(benzene-4,1diyl)tetrakis(aza))tetrakis (methan-1-yl-1-yliden) tetrabenzoic acid, H_4_MTA: triphenylmethanol (97%), aniline (99.5%), ethyl 4-aminobenzoate (benzocaine, ≥98%), oxone (≥99%), Pd/C (10% Pd basis), RANEY^®^ Nickel aluminium alloy (50% Al basis, 50% Ni basis), hydrazine monohydrate (80% solution in H_2_O), sodium hydroxide (90%), sodium bicarbonate (99.5%), anhydrous sodium sulphate (99%), isopentyl nitrite (≥97%), hypophosphorus acid (50 wt% in H_2_O), ethanol (99.8%), methanol (extra dry, ≥99%), glacial acetic acid (≥99%), tetrahydrofufan (99%), dichloromethane (99.5%), acetic anhydride (≥98%), hydrochloric acid (36%), fuming acid (99% HNO_3_), sulphuric acid (97%) and amorphous porous materials: zinc(ii) nitrate hexahydrate (≥98%), cadmium(ii) nitrate tetrahydrate (98%), *N*,*N*′-dimethylformamide (≥99%) were obtained from Sigma-Aldrich, Acros Organics or eMolecules companies, and used without further purification.

#### 2.1.2 Synthesis of H_4_MTA

**Fig. 1:**
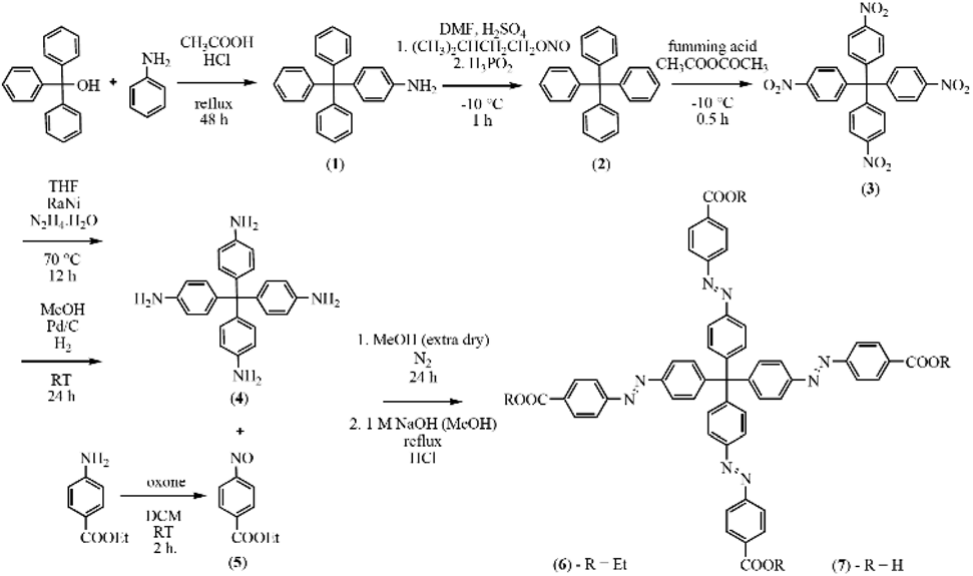
Synthesis of methanetetrayltetrakis(benzene-4,1-diyl)tetrakis(aza)tetrakis(methan-1-yl-1-yliden)tetrabenzoic acid (H_4_MTA) with corresponding synthetic conditions.

H_4_MTA linker was prepared by seven-step organic synthesis according to the reactions presented in Fig. 1 and procedures described below. Reaction conditions were chosen and modified from the literature procedures.^46–49^

##### 2.1.2.1 Synthesis of 4-tritylaniline (1)

4-Tritylaniline was prepared by the condensation reaction of triphenylmethanol and aniline. Triphenylmethanol (20 g; 77 mmol) was dissolved in glacial acetic acid (230 cm^3^) and to this solution aniline (11 cm^3^; 121 mmol) was added. Finally, the reaction mixture was acidified with concentrated HCl (38.5 cm^3^) and refluxed at 130 °C for 48 h. Subsequently, the reaction mixture was cooled to ambient temperature and diluted in water (1.5 dm^3^). The resulting precipitate of 1 was filtered off under reduced pressure using a Buchner funnel. The filter cake was washed several times with water to remove residual acetic and hydrochloric acid. The washed product was placed in an oven and dried at 70 °C overnight to yield 18.16 g of 1 (71% based on triphenylmethanol). ^1^H NMR (600 MHz, CDCl_3_) *δ* (ppm): 5.00 (s, 2H); 6.46 (d, 2H, *J* = 6.98 Hz); 6.75 (d, 2H, *J* = 6.98 Hz); 7.09–7.29 (m, 15H) ppm. Elemental analysis for C_25_H_21_N_1_ (335.44 g mol^−1^) calculated: C, 89.51%; H, 6.31%; N, 4.18%; measured: C, 89.46%; H, 6.21%; N, 4.09. IR (KBr): *ν*(NH) 3469 (w), 3382 (w); *ν*(CH)_ar_. 3082 (w), 3054 (w), 3028 (w); *δ*(NH) 1621 (m); *ν*(C=C)_ar_. 1593 (w), 1511 (w); *δ*(CCH)_ar_. 1183 (w), 1081 (w), 1033 (w); *γ*(CCH)_ar_. 825 (m) cm^−1^.

##### 2.1.2.2 Synthesis of tetrakis(phenyl)methane (2)

The tetrakis(phenyl)methane core was prepared by deamination reaction of 4-tritylaniline. 1 (10 g; 30 mmol) was dispersed in *N*,*N*′-dimethylformamide (DMF, 125 cm^3^) and the solution was acidified by dropwise addition of concentrated sulphuric acid (62.5 cm^3^). Sulphuric acid protonated the primary amine of 1, causing its complete dissolution in DMF. Subsequently, the reaction mixture was cooled with the solution of ice/sodium chloride to ≈−10 °C, and then isopentyl nitrite (15 cm^3^; 112.5 mmol) was added dropwise. After the addition of isopentyl nitrite, the solution was stirred for 1 h at ≈−10 °C. Hypophosphorous acid (30 cm^3^; 0.5 mol) was added dropwise after the mentioned reaction time, which catalysed the azo bond cleavage of the intermediate. During the addition of H_3_PO_2_, the release of nitrogen was observed and a precipitate of 2 was formed in the mixture. 2 was filtered off under reduced pressure, washed with DMF, methanol and dried in an oven at 70 °C overnight (yield: 8.83 g, 92.4% based on 1). ^1^H NMR (600 MHz, CDCl_3_) *δ* (ppm): 7.21 (m, 20H) ppm. ^13^C NMR (151 MHz, CDCl_3_) *δ* (ppm): 67.0; 125.8; 127.4; 131.1; 146.7 ppm. Elemental analysis for C_25_H_20_ (320.43 g mol^−1^) calculated: C, 93.71%; H, 6.29%; measured: C, 92.67%; H, 6.19%. IR (KBr): *ν*(CH)_ar._ 3080 (w), 3052 (w), 3029 (w), 3011 (w); *ν*(C=C)_ar._ 1591 (w), 1490 (m); *δ*(CCH)_ar._ 1181 (w), 1080 (w), 1034 (w); *γ*(CCH)_ar._ 748 (s), 699 (s) cm^−1^.

##### 2.1.2.3 Synthesis of tetrakis(*p*-nitrophenyl)methane (3)

Tetrakis(*p*-nitrophenyl)methane was prepared by nitration reaction of 2 using fuming acid. Fuming acid (nitric acid, 99% HNO_3_, 12.81 cm^3^; 0.3 mol) was cooled to ≈−10 °C using H_2_O/NaCl bath and 2 (2.56 g; 8 mmol) was added to the solution in small portions as the reaction is extremely exothermic. After complete addition of 2, acetic anhydride (4.27 cm^3^; 45 mmol) was added dropwise and the reaction mixture was stirred for 30 min. The last step was a dilution of the reaction mixture into glacial acetic acid (17.1 cm^3^). A yellow precipitate of 3 was filtered off, washed with acetic acid, methanol and dried in an oven at 50 °C overnight (yield 1.63 g, 40.8% based on 2). Finally, the product was recrystallised from toluene, resulting in a reaction yield of 30%. ^1^H NMR (600 MHz, DMSO): *δ* = 7.61 (8H, d, *J* = 9.01 Hz); 8.22 (8H, d, *J* = 9.01 Hz) ppm. Elemental analysis for C_25_H_16_N_4_O_8_ (500.42 g mol^−1^) calculated: C, 60.00%; H, 3.22%; N,11.20%; N, 11.20%; measured C, 60.31%; H, 3.18%; N, 11.02%. IR (KBr): *ν*(CH)_ar._ 3109 (w), 3079 (w); *ν*(C=C)_ar._ 1604 (m), 1589 (m), 1493 (w); *ν*_as._(C–NO_2_) 1520 (s); *ν*_s._(C–NO_2_) 1337 (s); *δ*(CCH)_ar._ 1106 (m), 1009 (w); *γ*(CCH)_ar._ 837 (s), 743 (s) cm^−1^.

##### 2.1.2.4 Preparation of tetrakis(*p*-aminophenyl)methane (4)

The aim of this synthetic step was the reduction reaction of nitro to an amino group. The reduction was carried out by two synthetic procedures using different catalysts and reducing agents: hydrazine/RaNi or Pd/C and hydrogen, respectively.

###### 2.1.2.4.1 Synthesis using hydrazine/RaNi

The reaction mixture of 3 (0.3 g; 0.9 mmol), hydrazine monohydrate (N_2_H_4_·H_2_O; 0.4 g; 8 mmol; 50% aqueous solution) and RANEY^®^ nickel (Ra/Ni; 10 g) in THF (50 cm^3^) was refluxed for 12 hours. The reaction mixture was hot filtered under reduced pressure to remove the heterogeneous catalyst and washed with THF. The filtered solution was evaporated under vacuum until the first visible crystals of the product were formed. The crystallised product 4 was filtered off, slightly washed with ethanol and finally purified from hot ethyl acetate (yield: 0.18 g, 78% based on 3).

###### 2.1.2.4.2 Synthesis using Pd/C and hydrogen

3 (0.5 g; 0.99 mmol) was dissolved in methanol (20 cm^3^) and heterogeneous Pd/C catalyst (0.1 g; 0.05 mmol) was added to the solution in small portions. The reaction mixture was stirred vigorously and the hydrogen atmosphere was created as follows: the pressure in the system was reduced by a vacuum pump until boiling of the methanol occurred and hydrogen was introduced into the system. This procedure was repeated four times. Subsequently, the reaction mixture was stirred for 24 h in a reducing atmosphere of hydrogen at room temperature. The product was filtered off, washed with methanol and dried under vacuum. 4 was purified by column chromatography on silica gel using a solvent mixture of DCM : MeOH (7 : 1, v/v) as mobile phase (yield: 0.27 g, 71% based on 3).

###### 2.1.2.4.3 ^1^H NMR (600 MHz, DMSO) *δ* (ppm)

4.84 (4H, s); 6.39 (8H, d, *J* = 8.4 Hz); 6.67 (8H, d, *J* = 8.4 Hz). Elemental analysis for C_25_H_24_N_4_ (380.48 g mol^−1^) calculated: C, 78.92%; H, 6.36%; N, 14.73%; measured: C, 79.13%; H, 6.28%; N, 14.49%. IR (KBr): *ν*(NH_2_) 3465 (m), 3382 (m); *ν*(CH)_ar._ 3082 (w), 3054 (w), 3027 (w); *ν*(C=C)_ar._ 1505 (s), 1593 (m); *δ*(NH_2_) 1621 (m); *ν*(C–N) 1286 (s); *δ*(CCH)_ar._ 1182 (m), 1115 (m), 1033 (w); *γ*(CCH)_ar._ 825 (w), 749 (m), 701 (s) cm^−1^.

##### 2.1.2.5 Preparation of ethyl 4-nitrosobenzoate (5)

2.5 g (15 mmol) of ethyl 4-aminobenzoate was dissolved in 50 cm^3^ of dichloromethane (DCM) and solution of oxone (potassium peroxymonosulfate, KHSO_5_·0.5KHSO_4_·0.5K_2_SO_4_) 20.3 g (66 mmol) in 200 cm^3^ of deionised water was added. The reaction mixture was stirred at room temperature for 2 h. After the colour change from colourless to green, the mixture was extracted with CH_2_Cl_2_ and washed with H_2_O several times. The organic layer was further washed with 30 cm^3^ of 1 M HCl and 40 cm^3^ of NaHCO_3_ saturated aqueous solution. The suspension was extracted with CH_2_Cl_2_ (4 × 50 cm^3^) and dried with Na_2_SO_4_. The organic layers were collected and the solvent was removed under reduced pressure to form the light-yellow product of 5 (yield 2.03 g, 75% based on ethyl 4-aminobenzoate). ^1^H NMR (600 MHz, CDCl_3_) *δ* (ppm): 1.24 (3H, t, *J* = 7.1 Hz), 4.14 (2H, q, *J* = 7.1 Hz), 8.07 (2H, ddd, *J* = 8.5, 1.6, 0.5 Hz), 8.13 (2H, ddd, *J* = 8.5, 1.4, 0.5 Hz). Elemental analysis for C_9_H_9_NO_3_ (179.17 g mol^−1^) calculated: C, 60.33%; H, 5.06%; N, 7.82%; measured: C, 60.54%; H, 4.99%; N, 7.89%. IR (KBr): *ν*(CH)_ar._ 3103 (w), 3072 (w), 3050 (w); *ν*(CH)_aliph._ 2864 (w), 2901 (w), 2932 (w), 2979 (w); *ν*(C=O) 1703 (s); *ν*(C–N) 1529 (w); *γ*(CCH)_ar._ 874 (s), 689 (s) cm^−1^.

##### 2.1.2.6 Preparation of methanetetrayltetrakis(benzene-4,1-diyl)tetrakis(aza))tetrakis (methan-1-yl-1-yliden)tetrabenzoic acid ethyl ester (Et_4_MTA) (6)

Et_4_MTA was prepared by the condensation reaction of ethyl 4-nitrosobenzoate and tetrakis(*p*-aminophenyl)methane. Solution of 5 (1.6 g, 9 mmol) in 40 cm^3^ of acetic acid was added dropwise during 10 min time period in solution of 4 (0.7 g, 1.8 mmol) dissolved in 40 cm^3^ of glacial acetic acid and 50 cm^3^ of extra dry methanol under nitrogen. The reaction mixture was stirred at laboratory temperature in an inert atmosphere of nitrogen for 2 days. Formed orange suspension of 6 was filtered off, washed with saturated aqueous solution of NaHCO_3_, water and methanol (3 × 15 cm^3^) and dried at 60 °C in an oven overnight (yield: 1.21 g, 64% based on 4). ^1^H NMR (600 MHz, CDCl_3_) *δ* (ppm): 8.19 (8H, d, *J* = 8.9, H-3′′,5′′), 7.94 (8H, d, *J* = 8.9, H-2′′,6′′), 7.92 (8H, d, *J* = 8.8, H-3′,5′), 7.54 (8H, d, *J* = 8.8, H-2′,6′), 4.42 (8H, q, *J* = 7.1, H-5), 1.43 (12H, t, *J* = 7.1, H-6); ^13^C NMR (151 MHz, CDCl_3_) *δ* (ppm): 166.1 (C-4), 155.2 (C-1′′), 151.0 (C-4′), 149.3 (C-1′), 132.5 (C-4′′), 131.8 (C-2′,6′), 130.7 (C-3′′,5′′), 122.9 (C-3′,5′), 122.8 (C-2′′,6′′), 65.6 (C-1), 61.4 (C-5), 14.5 (C-6). Elemental analysis for C_61_H_52_N_8_O_8_ (1025.11 g mol^−1^) calculated: C, 71.47%; H, 5.11%; N, 10.93%; measured: C, 71.37%; H, 5.03%; N, 11.18%. IR (KBr): *ν*(C=O) 1679 (s); *ν*(C=C)_ar._ 1599 (w); *ν*(N=N) 1494 (w); *δ*(CCH)_ar._ 1085 (m), 1009 (m); *γ*(CCH)_ar._ 868 (m), 699 (s) cm^−1^. Assignments of hydrogen and carbon atoms are depicted in Fig. S1 in ESI† and 2D NMR experiments: ^1^H,^1^H–COSY; ^1^H,^13^C-HSQC; ^1^H,^13^C-HMBC spectra of 6 are presented in Fig. S2–S4 in ESI.†

##### 2.1.2.7 Preparation of methanetetrayltetrakis(benzene-4,1-diyl)tetrakis(aza))tetrakis(methan-1-yl-1-yliden)tetrabenzoic acid (H_4_MTA) (7)

The last synthesis step in H_4_MTA preparation was deesterification reaction of its ethyl ester (6). 6 (270 mg, 0.26 mmol) was suspended and stirred in 150 cm^3^ solution of 1 M NaOH in methanol and heated at 80 °C for 2 h. After this time the reaction mixture was cooled down to ambient temperature, filtrated and the solvent was removed under vacuum. The crude product was dissolved in water and solution was subsequently acidified with concentrated HCl until pH = 1. Orange solid of 7 was precipitated, filtered off, washed with water and dried in an over at 50 °C overnight (yield: 226 mg, 94% based on 6). ^1^H NMR (600 MHz, DMSO-d_6_) *δ* (ppm): 13.25 (4H, s, OH), 8.14 (8H, d, *J* = 8.5, H-3′′,5′′), 7.95 (8H, d, *J* = 8.5, H-2′′,6′′), 7.96 (8H, d, *J* = 8.8, H-3′,5′), 7.60 (8H, d, *J* = 8.5, H-2′,6′). ^13^C NMR (151 MHz, DMSO-d_6_) *δ* (ppm): 166.6 (C-4), 154.4 (C-1′′), 150.1 (C-4′), 149.2 (C-1′), 132.9 (C-4′′), 131.5 (C-2′,6′), 130.7 (C-3′′,5′′), 122.8 (C-3′,5′), 122.5 (C-2′′,6′′), 65.2 (C-1). Elemental analysis for C_53_H_36_N_8_O_8_ (912.9 g mol^−1^) calculated: C, 69.73%; H, 3.97%; N, 12.27%; measured: C, 69.35%; H, 4.08%; N, 12.18%. IR (KBr): *ν*(OH)_ar._ 3420 (w, br); *ν*(CH)_ar._ 3106 (w), 3069 (w); *ν*(CH)_aliph._ 2982 (w), 2960 (w), 2929 (w); *ν*(C=O) 1709 (s); *ν*(C=C)_ar._ 1597 (w); *ν*(N=N) 1521 (w); *δ*(CCH)_ar._ 1085 (m), 1009 (m); *γ*(CCH)_ar._ 858 (m), 697 (s) cm^−1^. 2D NMR experiments: ^1^H,^13^C-HSQC and ^1^H,^13^C-HMBC spectra of 7 are presented in Fig. S5 and S6 in ESI.†

#### 2.1.3 Synthesis of UPJS-13 and UPJS-14

**Fig. 2:**
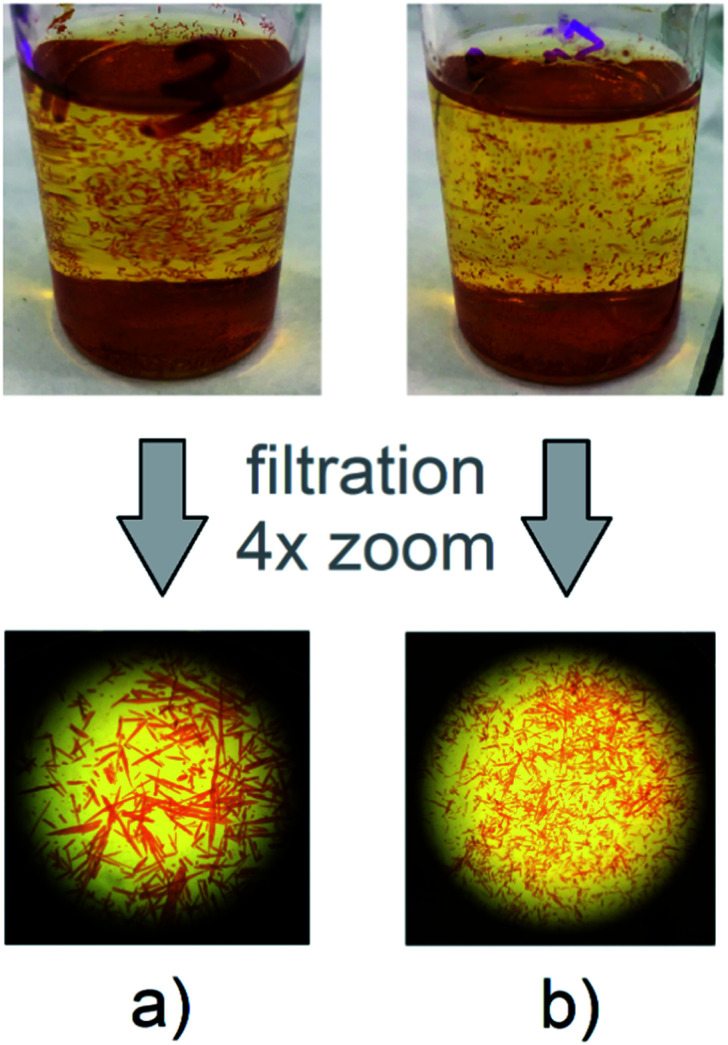
Prepared orange quasi-crystals of (a) UPJS-13 (AS) and (b) UPJS-14 (AS) in screwcapped vials and after filtration process and 4× zoom.

A mixture of H_4_MTA (10 mg, 0.011 mmol) and Cd(NO_3_)_2_·4H_2_O (27 mg, 0.088 mmol) or Zn(NO_3_)_2_·6H_2_O (6.5 mg, 0.022 mmol) were dissolved in a solvent mixture of DMF/H_2_O (6 cm^3^/0.5 cm^3^) in a screwcapped vial. The vial was capped and placed in an oven and reaction mixtures were heated at 80 °C for five days. Prepared orange crystals (see Fig. 2) of materials were filtered off, slightly washed with ethanol and dried in the stream of air (yield: 12.4 mg, 85% for UPJS-13 (AS) and 14.8 mg, 89% for UPJS-14 (AS) based on H_4_MTA). UPJS-13 (AS), {[Zn_2_(MTA)]·4H_2_O·3DMF}_*n*_, *M*_r_ = 1331.00 g mol^−1^, CHN and AAS: clcd: C 55.95%, H 4.62%, N 11.58%, Zn 9.83%; exp.: C 55.84%, H 4.66%, N 11.69%, Zn 9.95%; UPJS-14 (AS), {[Cd_2_(MTA)]·5H_2_O·4DMF}_*n*_, *M*_r_ = 1516.15 g mol^−1^, CHN and AAS: clcd.: C 51.49%, H 4.65%, N 11.09%, Cd 14.83%; exp.: C 51.44%, H 4.61%, N 11.17%, Cd 14.76%.

#### 2.1.4 Preparation of the samples for gas adsorption measurements

##### 2.1.4.1 As-synthesized samples (AS)

The as-synthesized samples for nitrogen adsorption experiments were prepared by repeating the synthetic procedure described in Section 2.1.3 Synthesis of UPJS-13 and UPJS-14. After collecting of sufficient amount of the sample, the material was stored in the mother liquor. The collected material was further activated under vacuum at selected temperatures described in Section 2.2.6 Nitrogen adsorption measurements.

##### 2.1.4.2 Ethanol exchanged samples (EX)

The ethanol exchanged samples were prepared by immersing of the as-synthesized materials in ethanol for 14 days, while ethanol was changed every day. Ethanol exchanged samples were activated under vacuum at selected temperatures described in Section 2.2.6 Nitrogen adsorption measurements.

##### 2.1.4.3 Freeze-dried samples (FD)

**Fig. 3:**
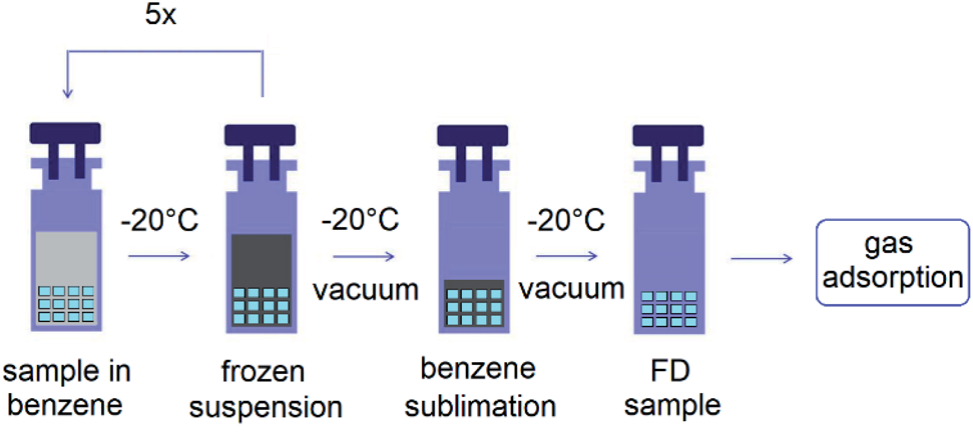
Scheme of the freeze-drying process.

The freeze-dried samples were prepared by immersing the as-synthesized materials in benzene for 14 days, while benzene was changed every day. Subsequently, the suspension of the sample in benzene was frozen at −20 °C. After five freeze–thaw cycles, the benzene exchanged material in sample cells were placed under vacuum using a turbomolecular pump at −20 °C for 24 h, and further heated under vacuum at selected temperatures described in Section 2.2.6 Nitrogen adsorption measurements. Schematic representation of the described freeze-drying process is shown in Fig. 3.

### 2.2 Methods and characterisation

#### 2.2.1 Elemental analysis and ICP-MS

The elemental analysis was performed using a CHNOS Elemental Analyzer Vario MICRO from Elementar Analysensysteme GmbH with a sample weight of approximately 3 mg. The determination of metal ions amount in as-synthesized aMOFs was carried out on the ICP-MS 7700 instrument developed by Agilent Technologies, in the atmosphere of argon. Before measurements, the samples were mineralised in hot aqua regia.

#### 2.2.2 Infrared spectroscopy

The infrared spectra of the samples were measured at ambient temperature and recorded using an Avatar FTIR 6700 spectrometer in the wavenumber range of 4000–400 cm^−1^ with 64 repetitions for a single spectrum. Materials were measured using KBr technique in mass ratio 1 : 100 (sample: KBr). Potassium bromide was before measurements dried in an oven at 500 °C for 4 h and cooled in a desiccator.

#### 2.2.3 NMR spectroscopy

**Fig. 4:**
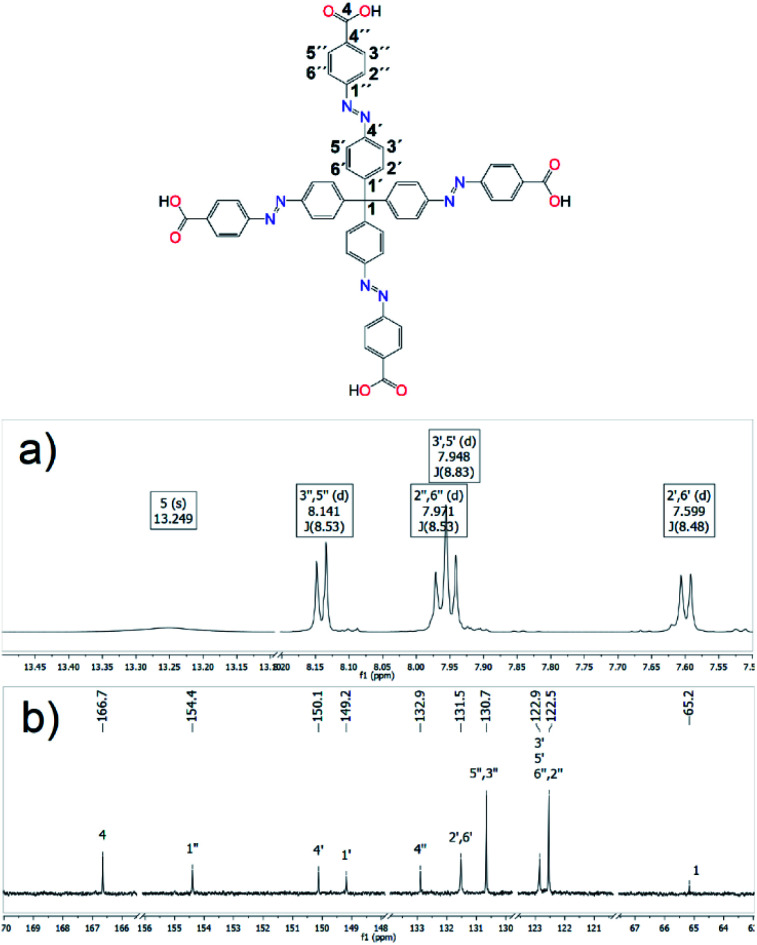
(a) ^1^H (600 MHz) and (b) ^13^C (151 MHz) NMR spectra of H_4_MTA in DMSO-d_6_ with assignments of hydrogen and carbon atoms.

Nuclear magnetic resonance data were collected on a Varian VNMRS 600 spectrometer operating at 599.87 MHz for ^1^H and 150.84 MHz for ^13^C. The mass concentration of all samples was approximately 10 mg/0.6 mL of DMSO-d_6_ or CDCl_3_, and the chemical shifts were referenced to the residual solvent peak (^1^H NMR 2.50 ppm and ^13^C NMR 39.5 ppm for DMSO-d_6_; ^1^H NMR 7.26 ppm and ^13^C NMR 77.0 ppm for CDCl_3_). The NMR data were recorded at 300 K, with chemical shifts (*δ*) reported in parts per million and coupling constants (*J*) in Hertz. The 2D experiments gCOSY, gHSQC and gHMBC were run using the standard Varian software. All data were analysed using MNova 7.1.1 (2012) software. The spectral acquisition parameters are summarised in Section 2.1.2 Synthesis of H_4_MTA and selected spectra are shown in Fig. 4 and S1–S7 in ESI.†

#### 2.2.4 Solid-state NMR spectroscopy

The solid-state NMR measurements of H_4_MTA and activated compounds, UPJS-13 and UPJS-14, were carried out on a Bruker Advance III HD WB/US NMR spectrometer at a temperature of 30 °C in a 4 mm probehead at a spinning frequency of 10 kHz (magnetic field strength 11.7T). In all cases powdered and dried samples were placed into zirconium(iv) oxide rotors with 4 mm size. ^13^C MAS NMR spectra (T_1_ filtered) of compounds were recorded using the single pulse experiment with a high power dipolar decoupling and a 2 × 10^3^ number of scans. A standard cross-polarization pulse (contact time ∼ 1 ms) sequence was used to measure ^13^C CP/MAS NMR spectra. The applied nutation frequency of B_1_(^1^H) and B_1_(^13^C) fields during the cross-polarization period was 62.5 kHz and the repetition delay was 2–4 s. Glycine was used as an external standard (176.03 ppm (C=O)) for calibration of ^13^C NMR scale.

#### 2.2.5 Thermal analysis

The thermal behaviour of prepared samples was studied by thermogravimetric analysis (TGA) with a sample weight of approximately 10 mg, using platinum crucibles. Samples were heated in the temperature range of 30–800 °C with a heating rate of 10 °C.min^−1^ in a dynamic air atmosphere with a flow rate of 50 cm^3^ min^−1^, using TGA Q600 apparatus.

#### 2.2.6 X-ray diffraction experiments

Single-crystal diffraction analysis were collected on a Bruker D8 VENTURE Kappa Duo PHOTON 100 diffractometer using MoKα radiation (*λ* = 0.71073 Å). The sample crystals were cooled to −150 °C by using an Oxford Cryostream cooler. Experimental data were processed by the diffractometer software. Powder X-ray diffraction (PXRD) experiments were done in reflection geometry using a Bruker D8 ADVANCE ECO multipurpose diffractometer. In order to achieve a parallel and clean X-ray beam for PXRD experiments, initially divergent Cu-Kα radiation (*λ* = 1.54056 Å) emitted from copper X-ray lamp was further guided through the multi-layered mirror and set of slits. Powder samples were loaded in a glass frame and PXRD experiments were done by 2*θ* continuous scan at 0.5° min^−1^ from 10° to 100° so that scattered photons were recorded every 0.02° using a NaI scintillation detector at ambient temperature.

#### 2.2.7 Gas adsorption measurements

Nitrogen adsorption isotherms were taken using a Quantachrome Nova 1200e apparatus at −196 °C. Prior to nitrogen adsorption, the samples were outgassed at different temperatures (60, 80, 100, 120, 150 and 200 °C) for 12 h under the vacuum to remove solvent molecules from the pores. The adsorption isotherms of desolvated samples were collected in a relative pressure range from *p*/*p*_0_ = 0.005 to 0.95. Based on the nitrogen adsorption measurements, the BET specific surface area (*S*_BET_) of each sample was evaluated using adsorption data in a *p*/*p*_0_ range from 0.05 to 0.20.

The gas adsorption measurements at 30 °C and pressures up to 20 bar were made with carbon dioxide and methane using a homemade high-throughput instrument.^50^ Gas adsorption was measured *via* a manometric gas dosing system on both samples in parallel. The amounts of gas adsorbed were calculated by an equation of state using the Reference Fluid Thermodynamic and Transport Properties (REFPROP) software package 8.0 of the National Institute of Standards and Technology (NIST).^51^ Approximately 100 mg of sample was used, and the sample was thermally activated individually *in situ* under a primary vacuum, at a given final temperature overnight. Methane was of 99.9995% purity (N55), carbon dioxide was of 99.995% purity (N45) and gases were obtained from Air Liquide. The weight of the samples used in the high-pressure adsorption experiments was approximately 100 mg and before measurements, the freeze-dried samples were activated at 80 °C under vacuum for 12 h.

## 3 Results and discussion

### 3.1 Synthesis and NMR spectroscopy

The H_4_MTA ligand used in the preparation of aMOFs in the present study was synthesized by the seven-step organic synthesis (see Fig. 1). First, a tetraphenylmethane core was synthesized by the condensation reaction of triphenylmethanol and aniline and the prepared product was subsequently deaminated. Further reactions led to the introduction of nitro groups using fuming acid, which were subsequently reduced to amine groups. Described reactions led to the preparation of the first essential component required for the construction of H_4_MTA. The second basic component, ethyl 4-nitrosobenzoate, was prepared by transforming the nitro group to the nitroso group using oxone. The resulting H_4_MTA acid was prepared by the condensation reaction of ethyl 4-nitrosobenzoate with tetrakis-(*p*-aminophenyl)methane in anhydrous methanol and prepared ethyl ester was further deesterified in a concentrated solution of sodium hydroxide in methanol, followed by acidifying the solution to pH = 1.

NMR spectroscopy was used for molecular structure determination of all prepared organic compounds (see 2.1.2 Synthesis of H_4_MTA). The procedure to assign the NMR data of derivative 6 is described as an example. Compound 6 consists of twelve carbons. The carbonyl carbon is easily detectable in ^13^C NMR spectrum because of the chemical shift 166.2 ppm. The carbon peak at 166.2 ppm showed long-range couplings to signals of proton at 4.42 ppm (q, *J* = 7.1 Hz), protons at 8.19 ppm (d, *J* = 8.8 Hz) and protons at 7.94 ppm (d, *J* = 8.8 Hz). The proton signal at 4.42 ppm was easily assigned as H-5 because of the splitting pattern as quartet and chemical shift of corresponding carbon peak at 61.4 ppm in the HSQC spectra. The chemical shift for H-6, methyl group, is shown as a triplet of splitting pattern at the lowest frequency at 1.43 ppm. The protons at 8.19 ppm were H-3′′,5′′. They correlated with 130.7 ppm of ^13^C peak at HSQC spectrum and were coupled to both C-2′′,6′′ and C-1′′ in the HMBC spectrum. The chemical shift for H-2′,6′ (7.54 ppm (d, *J* = 8.8 Hz)) was assigned by HMBC correlation with quaternary carbon C-1 (65.6 ppm). Protons H-3′,5′ (7.92 ppm (d, *J* = 8.8 Hz)) were determined from the COSY experiment. The C-4′ is long-range coupled to H-2′,6′. The chemical shift for C-1′ was assigned by HMBC correlation with H-3′,5′.

The successful synthesis of the resulting tetrahedral tetraazo-tetracarboxylic acid was confirmed by ^1^H and ^13^C NMR measurements and corresponding spectra with assignments of hydrogen and carbon atoms are depicted in Fig. 4 and 2D NMR ^1^H,^13^C-HSQC and ^1^H,^13^C-HMBC spectra are presented in Fig. S5 and S6 in ESI.† Derivative 7 has a carboxyl group adjacent to carbonyl carbon atom C-4. Carbonyl carbon C-4 was found to correlate with protons H-3′′,5′′ in HMBC spectrum. The chemical shift of C-3′′,5′′ was observed at 130.7 ppm. The protons H-2′′,6′′ were observed at 7.96 ppm, whereas the corresponding carbon appeared at 122.5 ppm. The protons H-2′′,6′′ exhibit long-range coupling to the quaternary carbon C-4′′ at 132.9 ppm and the protons H-3′′,5′′ are long-range coupled to C-1''. The ^1^H doublet at 7.59 ppm exhibits a long-range coupling to C-1 (65.2 ppm) in the HMBC spectrum suggesting this signal corresponds to H-2′,6′. In the HMBC spectrum H-2′,6′ also correlate with the carbon at 150.1 ppm, which is C-4′. This implies that the chemical shift for H-3′,5′ is 7.94 ppm. C-1′ with the chemical shift at 149.2 ppm displays a long-range coupling to the protons H-3′,5′. Measured NMR spectra correlate well with a molecular structure of desired tetrahedral linker.

H_4_MTA was used for the preparation of UPJS-13 (AS) and UPJS-14 (AS) using zinc(ii) and cadmium(ii) nitrates at different synthetic conditions. From the structural analysis point of view, our primary goal was to prepare single crystals of studied materials suitable for single-crystal X-ray diffraction experiments. We performed many syntheses (specific synthetic conditions are summarized in Table S1 in ESI†), in which we changed the synthetic conditions: from the molar ratio of reactants, H_4_MTB in the form of carboxylic acid or sodium salt, through reaction time, reaction temperature, different solvents and its volume ratios. The mentioned variable conditions led to the formation of predominantly powdered products, which were not suitable for single-crystal X-ray diffraction experiments. Although aMOFs UPJS-13 and UPJS-14 were prepared in the form of crystals (see Fig. 2) which at first sight appeared to be single crystals, the results of structural measurements (SXRD and PXRD) showed their amorphous character. The best synthetic conditions for the preparation of presented aMOF compounds were found in the mixture of solvent DMF/H_2_O at 80 °C. The solvent molecules, DMF and water located in the cavities of the prepared compounds were exchanged with ethanol (UPJS-13 (EX) and UPJS-14 (EX)). A third variant of the materials was prepared by the freeze-drying process using benzene (UPJS-13 (FD) and (UPJS-14 (FD)). All described aMOFs materials were subsequently studied by nitrogen adsorption to find the best textural properties and after finding the best activation conditions, the selected compounds were tested as adsorbents for greenhouse gases, carbon dioxide and methane, by high-pressure adsorption measurements.

### 3.2 Single crystal and powder X-ray diffraction experiments

**Fig. 5:**
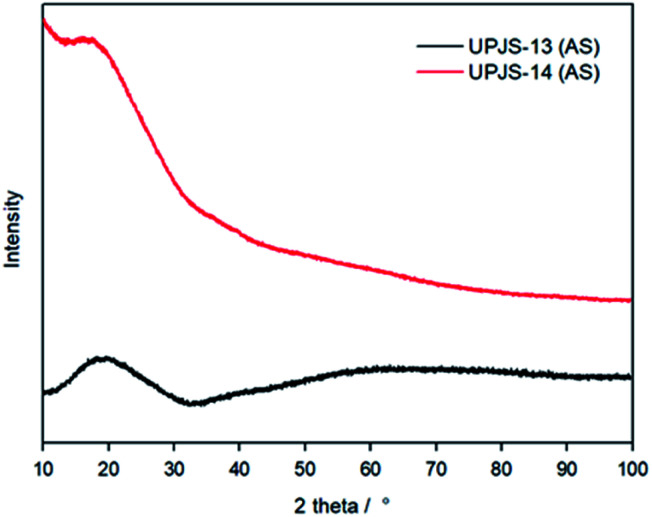
Powder X-ray diffraction patterns of UPJS-13 (AS) and UPJS-14 (AS).

Since the materials were prepared in the form of crystals (see Fig. 2), compounds were first subjected to single-crystal X-ray diffraction analysis to solve their crystal structures. Because the samples did not provide a diffraction image, even after selecting other crystals, the materials were subjected to powder X-ray diffraction (PXRD) experiments. These measurements aimed to confirm the amorphous composition of the whole bulk of prepared materials and confirm their amorphous character. Measured PXRD patterns of materials are shown in Fig. 5 and confirmed their disordered character. Aperiodic arrangements of atoms result in their PXRD patterns being dominated by broad “humps” caused by diffuse scattering and thus they are mostly indistinguishable from one another.

### 3.3 Infrared spectroscopy

**Fig. 6:**
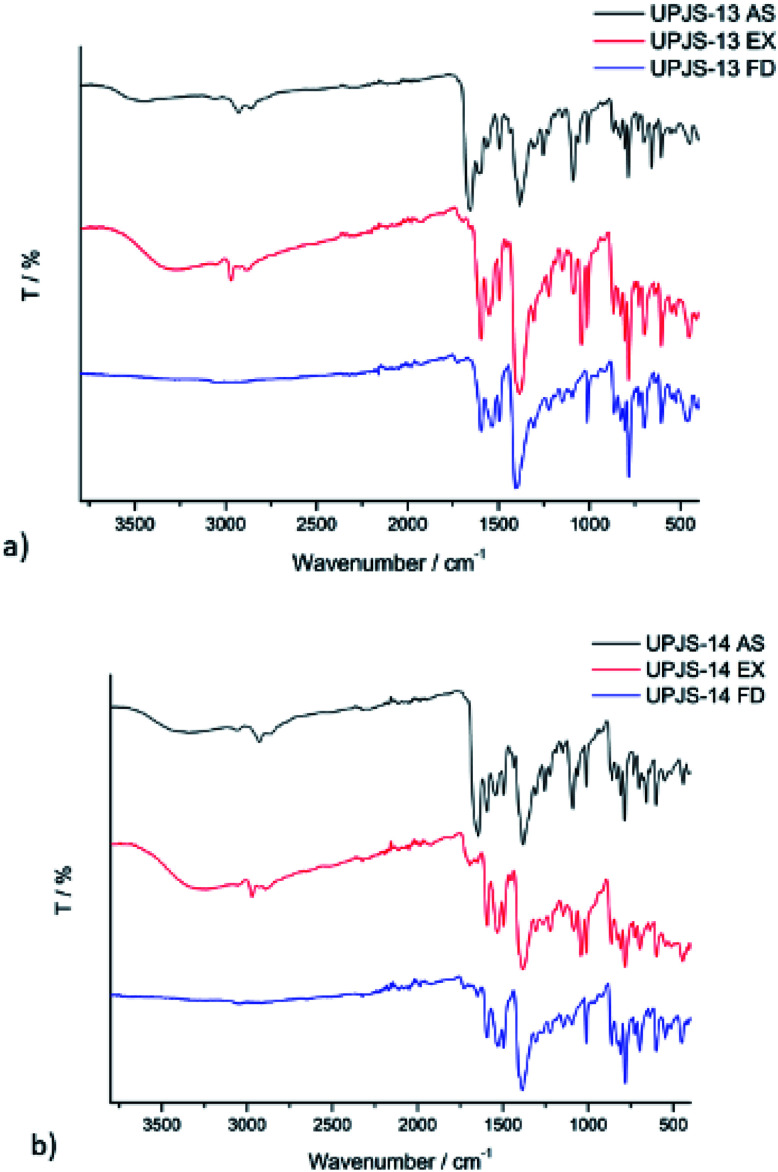
Infrared spectra of as-synthesized (AS), ethanol exchanged (EX) and freeze-dried (FD) materials of (a) UPJS-13 and (b) UPJS-14.

The infrared spectra of as-synthesized, ethanol exchanged and freeze-dried samples are shown in Fig. 6 and the assignment of absorption bands is summarised in Table 1. The IR spectra of both as-synthesized samples (see Fig. 6, black lines) exhibit broad band centred at about 3400 cm^−1^ which can be attributed to the OH stretching vibrations (*ν*(OH)) of water molecules, used in the synthesis. The presence of MTA linker in the prepared materials is evident from the intensive adsorption bands at 1603 cm^−1^ and 1378 cm^−1^ which can be assigned to antisymmetric and symmetric stretching vibrations of coordinated carboxylate groups (*ν*_as_(COO^−^) and *v*_s_(COO^−^)). The presence of MTA in prepared materials demonstrated absorption band at 1563 cm^−1^ for UPJS-13 (AS) and 1543 cm^−1^ for UPJS-14 (AS) which can be attributed to the N=N stretching vibrations of an azo bond. The stretching vibration of aromatic CH (*ν*(CH)_ar_) groups of MTA was found at 3053 cm^−1^ for UPJS-13 (AS) and 3047 cm^−1^ for UPJS-14 (AS). The stretching vibrations of aliphatic CH groups (*ν*(CH)_aliph_) of DMF molecules adsorbed in the porous system of samples were observed in the region under 3000 cm^−1^ (2926 and 2853 cm^−1^ for UPJS-14 (AS); 2929 and 2850 cm^−1^ for UPJS-14 (AS)). The presence of DMF is also evident from the strong absorption band of carbonyl group *ν*(C=O) located at 1656 cm^−1^ and the *ν*(C–N) vibration at 1253 cm^−1^ for both compounds. In the IR spectra of ethanol exchanged samples (see Fig. 6, red lines) absorption bands belonging to the MTA linker are still present, but the characteristic vibration *ν*(C=O) around 1650 cm^−1^ for DMF molecules is no longer present. Described observation confirms the successful exchange of DMF molecules for ethanol. Ethanol molecules in the IR spectra showed a shift in *ν*(OH) vibration to lower wavelength values (3277 cm^−1^ for UPJS-13 (EX) and 3260 cm^−1^ for UPJS-14 (EX)) and also new aliphatic *ν*(CH)_aliph_ vibrations appeared under 2930 cm^−1^. In the IR spectra of freeze-dried compounds (see Fig. 6, blue lines), no characteristic absorption bands originated from DMF or ethanol are present, which confirm the successful activation of UPJS-13 and UPJS-14 materials.

**Table 1:** Assignment of vibrations and corresponding wavenumbers to characteristic absorption bands in IR spectra of prepared and pretreated materials^a^

	UPJS-13 (AS)	UPJS-13 (EX)	UPJS-13 (FD)	UPJS-14 (AS)	UPJS-14 (EX)	UPJS-14 (FD)
*ν*(OH)	3440 (w, br)	3277 (w, br)	—	3407 (w, br)	3260 (w, br)	—
*ν*(CH)_ar_	3053 (w)	3053 (w)	—	3047 (w)	3047 (w)	—
*ν*(CH)_aliph_	2926 (w)	2926 (w)	—	2929 (w)	2929 (w)	—
	2853 (w)	2853 (w)		2850 (w)	2850 (w)	
*ν*(C=O)	1656 (s)	—	—	1656 (s)	—	—
*ν*(N=N)	1563 (w)	1563 (w)	1563 (w)	1543 (w)	1543 (w)	1543 (w)
*ν*_as_(COO^−^)	1603 (s)	1603 (s)	1603 (s)	1603 (s)	1603 (s)	1603 (s)
*ν*(CC)_ar_	1490 (m)	1490 (m)	1490 (m)	1493 (m)	1493 (m)	1493 (m)
*ν*_as_(COO^−^)	1378 (s)	1378 (s)	1378 (s)	1378 (s)	1378 (s)	1378 (s)
*ν*(CN)	1253 (m)	—	—	1253 (m)	—	—
*δ*(COO^−^)	785 (s)	785 (s)	785 (s)	787 (s)	787 (s)	787 (s)

a w – weak, m – medium, s – strong, br – broad, ar – aromatic, aliph – aliphatic, s – symmetric, as – antisymmetric.

### 3.4 Thermal stability study

**Fig. 7:**
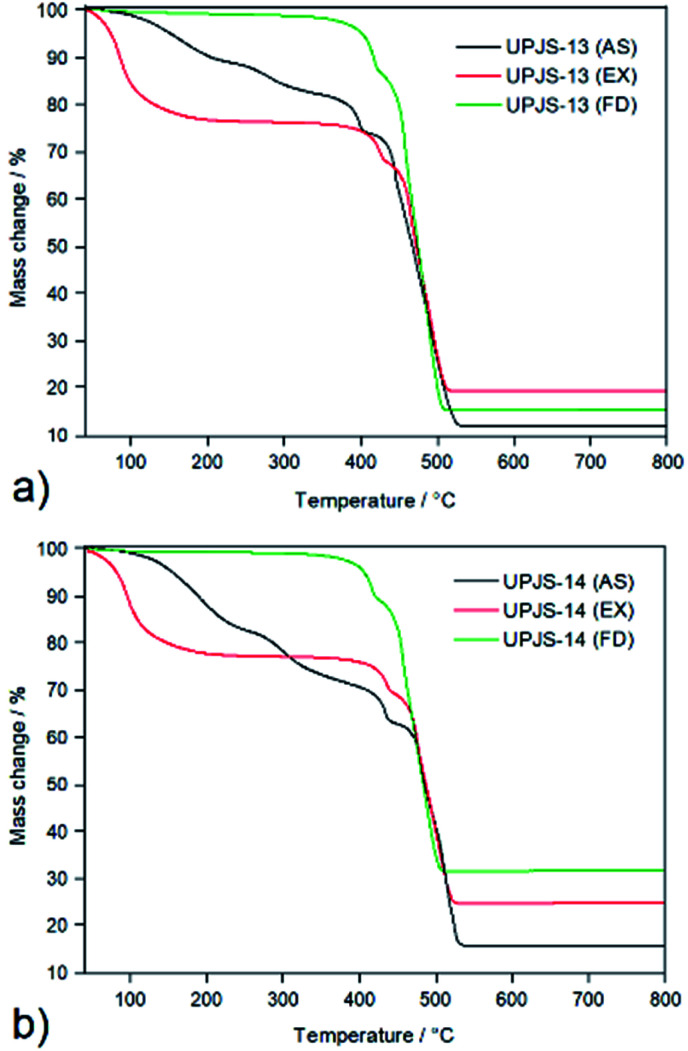
Thermogravimetric curves of as-synthesized, ethanol exchanged and freeze-dried samples of (a) UPJS-13 and (b) UPJS-14 measured in an air atmosphere and temperature range of 30–800 °C.

The thermal behaviour and stability of as-synthesized, ethanol exchanged and freeze-dried compounds were investigated using thermogravimetry and obtained TG curves are shown in Fig. 7. The AS samples (see black curves in Fig. 7) are thermally stable after heating to 80 °C, above this temperature in the temperature range 80–380 °C desolvation processes take place in two overlapping decomposition steps. In mentioned range total mass loss of 21.6 wt% for UPJS-13 (AS) and 32.0 wt% for UPJS-14 (AS) was observed and correspond to release of solvent molecules (3 × DMF, 4 × H_2_O, clcd. mass loss 21.9 wt% for UPJS-13 (AS); 4 × DMF, 5 × H_2_O, clcd. mass loss 31.8 wt% for UPJS-14 (AS)). Further heating led to the decomposition of frameworks, which occurs in 380–530 °C. Final thermal decomposition products were ZnO (observed residual mass 12.6 wt%, clcd. residual mass 12.2 wt% for UPJS-13 (AS)) and CdO (observed residual mass 17.4 wt%, clcd. residual mass 16.9 wt% for UPJS-14 (AS)). The desolvation process in ethanol exchanged samples takes place at a lower temperature (see red curves in Fig. 7), the release of ethanol molecules starts at the beginning of heating and complete desolvation occurs about 180 °C. The desolvated forms of UPJS-13 (EX) and UPJS-14 (EX) are thermally stable from 180–420 °C as is seen from the plateau on TG curves. The decomposition of polymeric frameworks takes place in two overlapping decomposition steps, similarly to the as-synthesized samples. As can be seen from obtained TG curves for EX materials, the solvent exchange process significantly impacts the compounds' activation temperature. The TG curves of the freeze-dried samples (see green curves in Fig. 7) confirm the successful activation of the compounds, as no weight loss is observed in the temperature range 30–420 °C. The decomposition of UPJS-13 (FD) and UPJS-14 (FD) frameworks, [Zn_2_(MTA)] and [Cd_2_(MTA)], takes place in temperature range 420–530 °C with residual masses 15.5 wt% and 33.9 wt% corresponding to the formation of ZnO and CdO as final decomposition products (clcd. residual masses 15.7 wt% for ZnO and 34.4 wt% for CdO).

### 3.5 Solid-state NMR spectroscopy

**Fig. 8:**
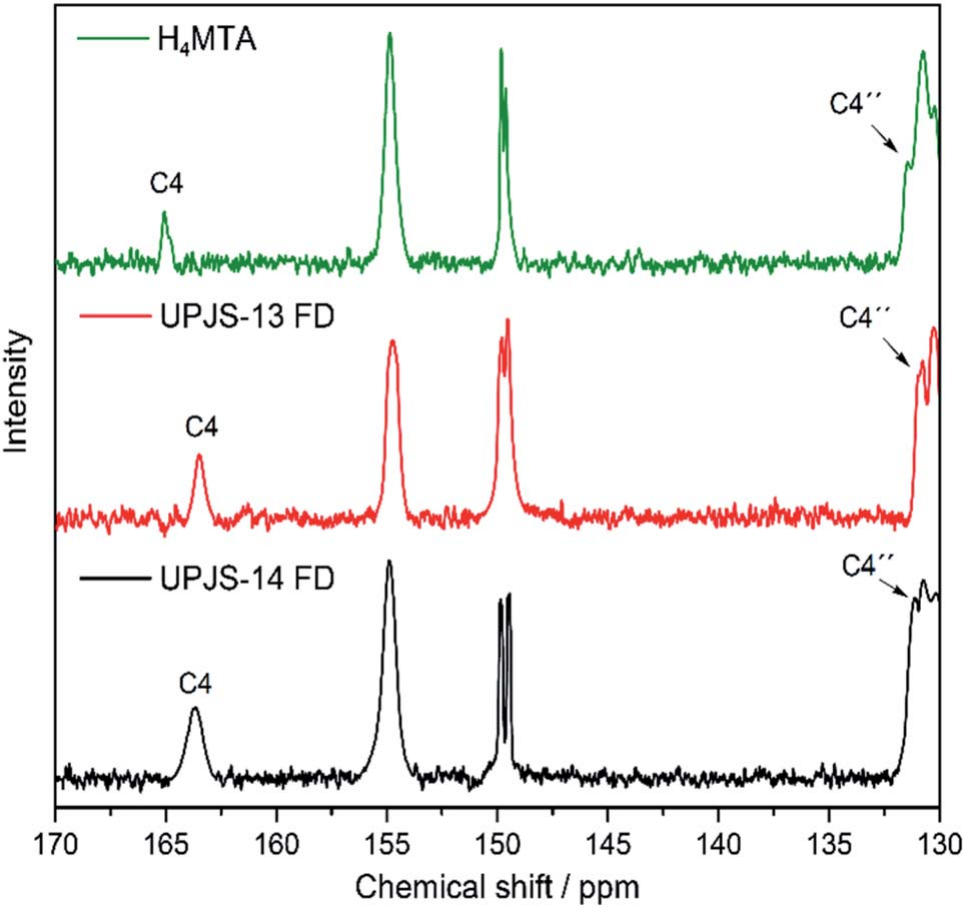
Assignment of the signals to the carbon atoms in H_4_MTA and activated forms of UPJS-13 and UPJS-14 in ^13^C CP/MAS NMR spectra.

The formation of interactions/bonds between the building blocks in the prepared aMOF materials was also studied by solid-state NMR (ssNMR) spectroscopy. A comparison of the ^13^C CP/MAS NMR spectra of the linker (H_4_MTA) and the freeze-dried materials UPJS-13 and UPJS-14 are shown in Fig. 8. The ^13^C NMR spectra of H_4_MTA measured in the liquid (see Fig. 4) and solid phases (see Fig. 8) show differences in the chemical shifts of the individual signals, which are mostly shifted in the solid phase to lower values. Changes in the position of the NMR signals can be explained by the formation of intermolecular interactions^52^ between H_4_MTA molecules in the solid-state. However, it should be noted that the particle size of the material used for measurement can also affect the position of the signals in the resulting NMR spectrum.^53^ Significant differences were observed for the carbonyl carbon (C4) signal, which shifts 2.6 ppm lower from the original value (166.7 ppm) in the liquid phase. This observation can be explained by the formation of dimers between the carboxyl groups of H_4_MTA molecules with each other.^54^ Significant changes in chemical shifts occur after coordination of the MTA linker to Zn(ii) and Cd(ii) ions, when significant changes in the signal positions of the carbon atoms C4 and C4′′ were observed (see Fig. 8). Since Zn(ii) and Cd(ii) ions are diamagnetic metals, they do not show so significant changes in chemical shifts after coordination of linker molecules compared to the paramagnetic ions.^55–58^ Nevertheless, based on the described changes of chemical shifts in the ^13^C CP/MAS NMR spectra of H_4_MTA and prepared compounds, the formation of a coordination bond between the building blocks can be confirmed.

### 3.6 Gas adsorption

#### 3.6.1 Nitrogen adsorption measurements

**Fig. 9:**
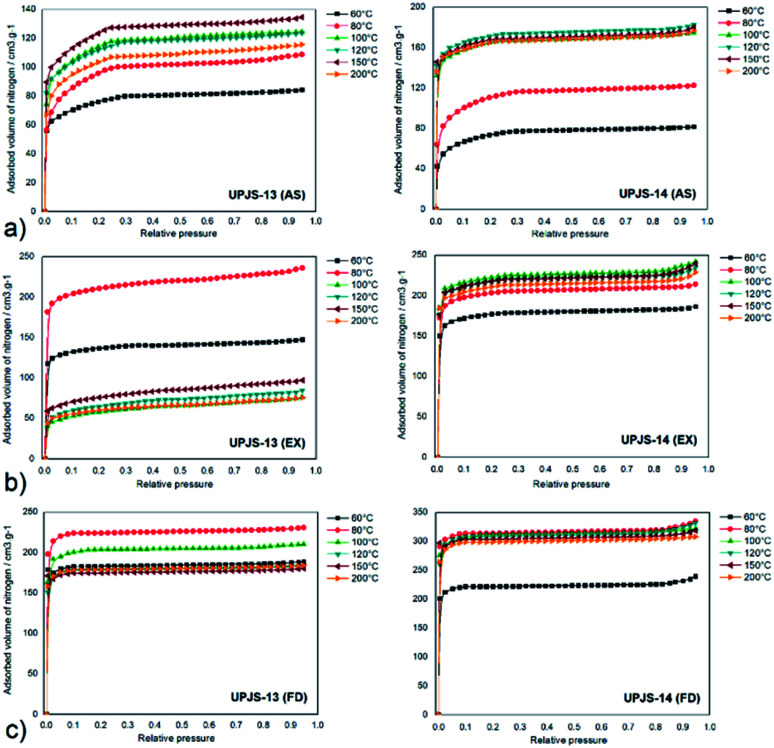
Nitrogen adsorption isotherms of (a) as-synthesized, (b) ethanol exchanged and (c) freeze-dried samples of UPJS-13 and UPJS-14 after activation at 60, 80, 100, 120, 150 and 200 °C.

**Fig. 10:**
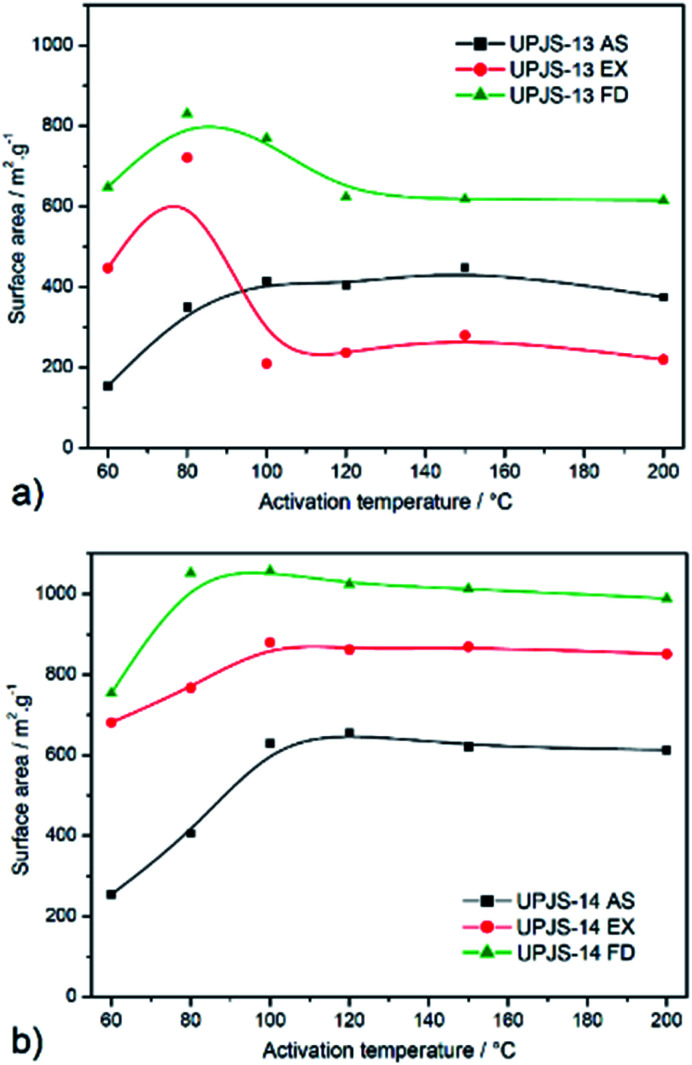
Calculated BET surface areas from nitrogen isotherms of activated materials at 60, 80, 100, 120, 150 and 200 °C for (a) UPJS-13 and (b) UPJS-14.

To investigate the porosity of prepared aMOFs, nitrogen adsorption measurements at −196 °C were performed. The adsorption measurements were realised on as-synthesized (AS), ethanol exchanged (EX) and freeze-dried (FD) samples. Ethanol exchanged samples were prepared by immersing of as-synthesized materials in ethanol. Freeze-dried samples were prepared after replacing high boiling point solvent (DMF, b.p. 153.0 °C) inside the channels of prepared materials for benzene (b.p. 80.1 °C, m.p. 5.5 °C). Samples were further frozen and benzene was removed under vacuum by sublimation. Samples prepared by the described procedure were thermally activated at different temperatures (60, 80, 100, 120, 150 and 200 °C) under vacuum for 12 h. Obtained adsorption isotherms are presented in Fig. 9 and calculated BET surface areas (*S*_BET_) are summarised in Table 2 and shown in Fig. 10. The nitrogen adsorption measurements revealed a type I isotherm according to IUPAC classification, typical for microporous materials. The small difference in the shape of measured and ideal isotherm could be explained by the presence of weaker attractive forces between nitrogen molecules with pore walls or by the nitrogen interaction with a variety of active surface sites and other vacant sites formed during the activation procedures (dislocation, defects, electron-rich azo group).

**Table 2:** The summary of calculated specific surface areas (*S*_BET_) for UPJS-13 and UPJS-14 after activation process at different temperatures

Sample	*S*_BET_ surface area (m^2^ g^−1^)					
	Activation temperature (°C)					
	60	80	100	120	150	200
**As-synthesized**						
UPJS-13 (AS)	154	349	415	404	448	375
UPJS-14 (AS)	254	405	629	656	621	613

**Exchanged**						
UPJS-13 (EX)	447	721	210	237	280	225
UPJS-14 (EX)	681	767	880	862	869	851

**Freeze dried**						
UPJS-13 (FD)	648	830	769	623	618	615
UPJS-14 (FD)	754	1057	1052	1024	1013	989

For as-synthesized materials containing DMF/water molecules in the pores, it is characteristic that as the activation temperature increases, the surface area gradually increases up to 100 °C (see black curves in Fig. 10a). Above this temperature, there is no more pronounced increase in *S*_BET_ and the values are almost constant. This is caused by the gradual removal of solvent molecules from the pore system of materials. The calculated surface area at 100 °C were 415 m^2^ g^−1^ for UPJS-13 (AS) and 629 m^2^ g^−1^ for UPJS-14 (AS). After activating samples at 200 °C, a slight decrease in surface area was observed (see Fig. 9, Table 2).

For UPJS-13 (EX), the trend in surface area values at selected activation temperature is different. The maximum *S*_BET_ of 721 m^2^ g^−1^ was reached at 80 °C and by subsequent heating at 100 °C decreases to the value of 210 m^2^ g^−1^. The further increase in temperature does not have a significant effect on the textural properties (see red curves in Fig. 10), probably due to the collapse of the polymer framework. Because ethanol has a low boiling point, its release at 100 °C occurs all at once, which lead to pore perforation associated with the destruction of the framework. The sample UPJS-14 (EX) showed similar behaviour compared to UPJS-14 (AS) and gradually increased the *S*_BET_ with increasing temperature up to 80 °C and the maximal surface area after activation at 100 °C was 880 m^2^ g^−1^.

The best textural properties were observed for materials after the freeze-drying process (see green curves in Fig. 10). The highest values of surface areas were calculated after activation of the samples at 80 °C (see green curves in Fig. 10, Table 2) and corresponding values were 830 m^2^ g^−1^ (pore volume, *V*_p_ = 0.57 cm^3^ g^−1^; pore diameter, *d* = 0.73 nm calculated by DFT) for UPJS-13 (FD) and 1057 m^2^ g^−1^ (pore volume, *V*_p_ = 0.72 cm^3^ g^−1^; pore diameter, *d* = 0.87 nm calculated by DFT) for UPJS-14 (FD). Gradual heating to 120 °C leads to a slight decrease in *S*_BET_ values and further heating did not affect the values of surface area for both compounds.

It is to note, that the observed *S*_BET_ values for UPJS-13 and UPJS-14 are higher compared with MOFs constructed from shorter tetrahedral linker, methanetetrabenzoate (MTB) containing Ca(ii) (126 m^2^ g^−1^),^59^ Zn(ii) (248 m^2^ g^−1^),^46^ Co(ii) (356 m^2^ g^−1^),^60^ Ba(ii) (358 m^2^ g^−1^),^59^ Cu(ii) (526 m^2^ g^−1^)^40^ and Ni(ii) (700 m^2^ g^−1^)^61^ ions. A further comparison could be performed with a copper(ii) MOF [Cu_2_(MTC)(H_2_O)_2_]·14DMF·5H_2_O,^40^ which contains similar extended tetrahedral linker MTC (methanetetra(biphenyl-*p*-carboxylate) to MTA described in the present study. The methanol exchanged and vacuum dried compounds, both activated at 60 °C exhibited surface area of 791 m^2^ g^−1^ and its freeze-dried form 1020 m^2^ g^−1^. The zinc(ii) analogue with formulae [Zn_2_(MTC)(H_2_O)_2_] may also be mentioned, which exhibited after dichloromethane exchange a surface area of 1170 m^2^ g^−1^^62^ and calculated *S*_BET_ is similar to UPJS-14 (FD). The highest reported surface area was published for zirconium(iv) MOF containing MTC linker with a composition of [Zr_6_(OH)_16_(MTC)_2_] and *S*_BET_ = 3411 m^2^ g^−1^.^63,64,74^

In summary, it can be concluded that UPJS-14 material showed better textural properties compared to UPJS-13, regardless of the post-synthetic treatment and activation conditions. The highest values of surface areas of prepared materials were observed for samples after the freeze-drying process, followed by ethanol-exchanged and as-synthesized samples. Compound UPJS-14 (FD) activated at 80 °C exhibited large surface area compared with other reported MOF compounds containing similar extended tetrahedral linker.

#### 3.6.2 High-pressure carbon dioxide and methane adsorption measurements

**Fig. 11:**
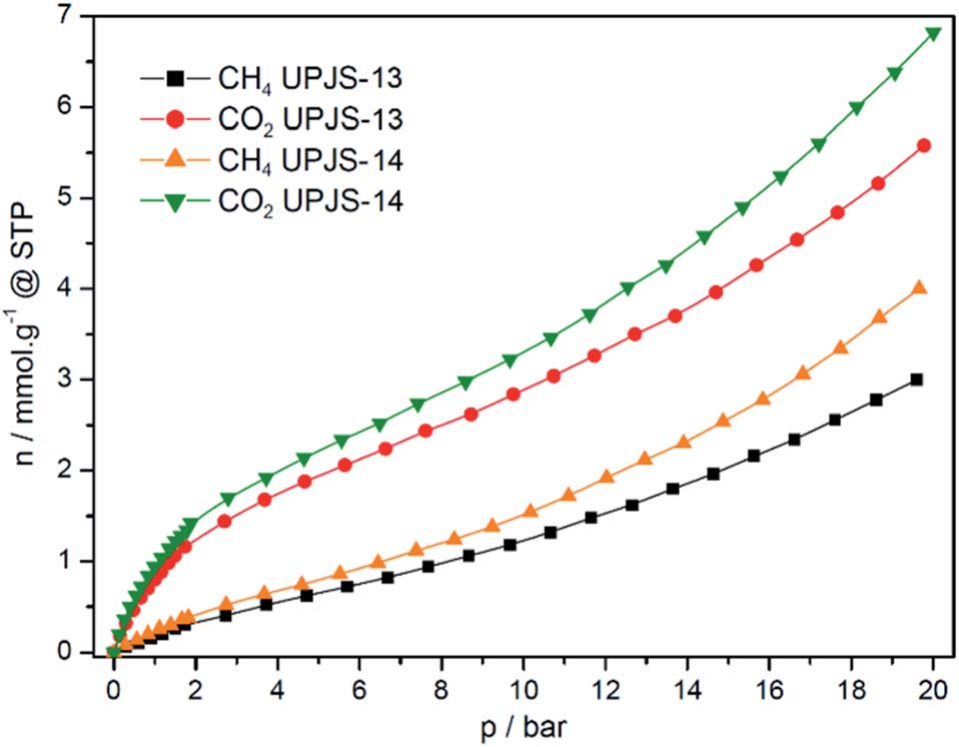
Methane and carbon dioxide high-pressure adsorption isotherms of UPJS-13 and UPJS-14 measured at 30 °C and pressure up to 20 bar.

As major components of greenhouse gases, carbon dioxide and methane have also been studied as probe molecules to investigate the sorption properties of prepared aMOFs. CO_2_ and CH_4_ high-pressure adsorption measurements were performed at 30 °C and pressure up to 20 bar on freeze-dried samples activated at 80 °C. Measured adsorption isotherms are shown in Fig. 11 and obtained maximal adsorption capacities at 1 and 20 bar are summarised in Table 3. The absence of district plateaus in the isotherms indicated that the maximal capacities were still not achieved and compounds are not saturated by the gases at 20 bar. Both selected probes are non-polar molecules with different quadrupole moments (0 C m^2^ for CH_4_, 14.3 × 10^40^ C.m^2^ for CO_2_), kinetic diameters (3.8 Å for CH_4_, 3.3 Å for CO_2_) and different boiling points (−161 °C for CH_4_, -78 °C for CO_2_) and described differences may influence the maximal adsorption uptake of gases.

**Table 3:** Adsorbed amount (in mmol g^−1^ and wt%) of CO_2_ and CH_4_ at 30 °C and pressure 1/20 bar in freeze-dried compounds after activation at 80 °C

	Bar	CO_2_		CH_4_	
		mmol g^−1^	wt%	mmol g^−1^	wt%
UPJS-13 (FD)	1	0.94	4.14	0.22	0.35
	20	6.82	30.1	2.21	1.4
UPJS-14 (FD)	1	0.80	3.52	0.36	0.58
	20	5.58	24.56	3.98	6.38

aMOFs storage capacities of carbon dioxide at 1 bar and 30 °C were 0.94 mmol g^−1^ (4.14 wt%) for UPJS-13 FD and 0.8 mmol g^−1^ (3.52 wt%) for UPJS-14 FD. With increasing pressure, an increase in the amount of adsorbed carbon dioxide was observed. The maximal adsorption uptake and corresponding adsorption capacities were 6.82 mmol g^−1^ (30.01 wt%) for UPJS-13 FD and 5.58 mmol g^−1^ (24.56 wt%) for UPJS-14 FD at 30 °C and 20 bar. Both compounds show high CO_2_ storage capacities, however, the UPJS-13 FD framework showed a higher affinity for CO_2_ compared to UPJS-14 FD, although it has a smaller surface area. Due to the presence of an electron-deficient carbon atom, carbon dioxide is expected to interact with protic electronegative functional groups, leading to strong chemisorption. This simple interaction has so far led to a number of nanoporous polymers in which nitrogen-rich functions are incorporated, such as triazine,^65,66^ tetrazole,^67^ imidazole,^68,69^ imide^70^ and amines.^42,71^ Increased CO_2_ adsorption capacity in UPJS-13 (FD) and UPJS-14 (FD) can be explained by an interaction between CO_2_ molecules and adsorption sites represented by a lone pair on the azo functional groups located in the molecular structure of the MTA linker. In the case of azo group, the interaction with CO_2_ was also confirmed by computational studies, in which the relative binding affinity of CO_2_ to the –N=N– group using *trans*-azobenzene as a model system were performed. Calculations have shown that the lowest energy structure of the CO_2_/*trans*-azobenzene complex indicates that carbon dioxide preferentially binds to the azo group of *trans*-azobenzene by physisorption with a binding energy of 17.0 kJ mol^−1^. CO_2_ also has a favourable interaction with the aromatic ring, including a large stabilizing energy, 14.4 kJ mol^−1^.^72^ It is known that MOFs with coordinatively unsaturated sites (CUSs) offer interesting possibilities for tuning the affinity of these materials towards certain adsorbates, potentially increasing their selectivity and storage capacity.^73^ In MOFs with CUSs, at least one available coordination site of the metal centre is occupied by an atom belonging to a solvent molecule. The solvent molecules can be removed through the activation process, leaving the metal atom with an open coordination site. Examples are Zn(ii) and Cd(ii) compounds, which have been constructed with methanetetrabenzoate (MTB, short tetrahedral carboxylate linker compared to MTA) with compositions: [Zn_2_(MTB)(H_2_O)_2_]·3H_2_O·3DMF^46^ and [Cd_4_(MTB)_2_(DMF)_4_]·4DMF·4H_2_O.^64,74^ The framework of zinc form imitates the PtS structure and contains paddle-wheel cluster (2 × CUSs, bold text in the formula) and the cadmium framework mimics the fluorite (CaF_2_) structure, which contains a tetranuclear cluster containing four terminally coordinated solvent molecules (4 × CUSs, bold text in the formula). We can only speculate, that the increased affinity of CO_2_ for the presented aMOFs is the presence of CUSs on Zn(ii) and Cd(ii) ions in the frameworks of UPJS-13 and UPJS-14.

According to the literature data, the high-pressure CO_2_ adsorption measurements were performed for two compounds containing MTB linker. Nickel(ii) MOF (*S*_BET_ = 700 m^2^ g^−1^) adsorbed 18.05 wt% (4.1 mmol g^−1^) of CO_2_ (ref. 61) and lead(ii) compound (*S*_BET_ = 980 m^2^ g^−1^) 19.81 wt% (4.5 mmol g^−1^) of CO_2_ (ref. 75 and 76) at 30 °C and 21 bar. The highest to date CO_2_ storage capacities were reported for the following unmodified MOFs: UiO(dpdc) (dpdc = 2,2′-bipyridine-5,5′-dimethyl, *S*_BET_ = 2646 m^2^ g^−1^)^77^ 18.11 mmol g^−1^ (79.7 wt%) at 30 °C and 20 bar; NU-111 (Cu(4-PmBC), 4-PmBC = 4-(pyrimidin-5-yl)benzoate, *S*_BET_ = 4932 m^2^ g^−1^)^78^ 14.04 mmol g^−1^ (61.8 wt%) at 25 °C and 30 bar; DGC-MIL-101 (DGC = dry gel conversion, Cr(BDC), BDC = benzene-1,4-dicarboxylate, *S*_BET_ = 4198 m^2^ g^−1^)^79^ 13.59 mmol g^−1^ (59.8 wt%) at 25 °C and 40 bar and DMOF (Zn_2_(BDC)_2_(DABCO), DABCO = 1,4-diazabicyclo[2.2.2]octane, *S*_BET_ = 1980 m^2^ g^−1^)^80^ 8.66 mmol g^−1^ (38.1 wt%) at 25 °C and 20 bar.

Methane is the main component of natural gas and the second most abundant greenhouse gas in Earth's atmosphere. CH_4_ is a non-polar molecule with zero quadrupole moment and has a low volumetric energy density, which poses a challenge for efficient storage to reduce its concentration in the atmosphere or for energy application. For this reason, methane was also used as an adsorbate for high-pressure adsorption and measured isotherms are presented in Fig. 11. Materials adsorb methane with a maximal storage capacity of 0.22 mmol g^−1^ (0.35 wt%) for UPJS-13 (FD) and 0.36 mmol g^−1^ (0.58 wt%) for UPJS-14 (FD) at 30 °C and 1 bar and 3.02 mmol g^−1^ (4.84 wt%) for UPJS-13 (FD) and 3.98 mmol g^−1^ (6.38 wt%) for UPJS-14 (FD) at 30 °C and 20 bar. It could be noted that the adsorbed amounts are approximately four/five times lower compared to carbon dioxide. Contrary, the values of stored CH_4_ are relatively high and can be explained by the pore size of aMOF materials. Several theoretical studies have shown that the optimum pore size for methane storage is 8 Å, which corresponds to distance between pore walls of about twice larger, than the molecule diameter.^81,82^ As it was described above, the effective pore size of UPJS-13 (FD) and UPJS-14 (FD) based on nitrogen adsorption are 7.3 and 8.7 Å; thus it could be an explanation for increased CH_4_ adsorption.

A comparison of the adsorption capacity with compounds containing shorter tetrahedral carboxylate linker (MTB) has shown that the CH_4_ adsorption capacities were reported for Ni(ii)^61^ and Pb(ii)^75^ compounds with a methane storage capacity of 3.29 wt% (2.05 mmol g^−1^) and 3.05 wt% (1.9 mmol g^−1^) at 30 °C and 21 bar, respectively. The highest documented CH_4_ capacities were reported for NU-125 (Cu(BTTI), BTTI = 5,5′,5′′-(4,4′,4′′-(benzene-1,3,5-triyl)tris(1H1,2,3-triazole-4,1-diyl))triisophthalate, *S*_BET_ = 3120 m^2^ g^−1^)^83^ which can store 11.47 mmol g^−1^ (18.40 wt%) at 25 °C and 20 bar and BUT-22 (Al_3_(O)(OH)(H_2_O)_2_(PPTTA)_3/2_, PPTTA = (1,4-phenylenebis(pyridine-4,2,6-triyl))tetrabenzoate, *S*_BET_ = 4380 m^2^ g^−1^)^84^ with storage capacity of 9.9 mmol g^−1^ (15.88 wt%) at 23 °C and 20 bar. Other MOFs, such as [Co(BDP)] (BDP = 1,4-benzenedipyrazolate, *S*_LANG_ = 2911 m^2^ g^−1^)^85^ and commercial product, Basolite A520 (aluminum fumarate, *S*_BET_ = 1300 m^2^ g^−1^)^86^ have also shown promise as methane sorbents with methane storage capacity of 6.5 mmol g^−1^ (10.43 wt%) at 25 °C and 20 bar and 3.80 mmol g^−1^ (6.10 wt%) at room temperature and pressure 20 bar, respectively.

## 4 Conclusion

The present study dealt with the preparation, characterisation and application of two novel aMOF compounds as adsorbents of greenhouse gases, carbon dioxide and methane. In the first phase, the extended nitrogen-rich tetraazo-tetracarboxylic acid, H_4_MTB was prepared by a seven-step organic synthesis. Spectroscopic and NMR measurements confirmed its successful preparation. The next step was the preparation of aMOF compounds in combination with H_4_MTB and zinc(ii) or cadmium(ii) cations under the formation of as-synthesized materials, UPJS-13 (AS) and UPJS-14 (AS). In order to facilitate the activation of the compounds, the solvent molecules located in the cavities of the as-synthesized materials were exchanged with ethanol (UPJS-13 (EX) and UPJS-14 (EX)). Another variant of the compounds was prepared by a freeze-drying process using benzene (UPJS-13 (FD) and UPJS-14 (FD)). The compounds were characterised by elemental analysis, ICP-MS, infrared spectroscopy, solid-state NMR spectroscopy and PXRD. All prepared aMOFs were studied by nitrogen adsorption to find the best activation conditions. The compounds showed the highest surface areas after the freeze-drying process and at an activation temperature of 80 °C (*S*_BET_ = 830 m^2^ g^−1^ for UPJS-13 (EX) and *S*_BET_ = 1057 m^2^ g^−1^ for UPJS-14 (EX)). aMOF materials were subsequently tested as greenhouse gases adsorbents, carbon dioxide and methane, by high-pressure adsorption measurements at 30 °C and pressure up to 20 bar. The maximal gas storage capacities were 30.01 wt% for UPJS-13 FD, 24.56 wt% for UPJS-14 FD of CO_2_ and 4.84 wt% for UPJS-13 FD and 6.38 wt% for UPJS-14 FD of CH_4_ at 30 °C and 20 bar. The increased adsorption capacity of carbon dioxide can be explained by the interaction of CO_2_ molecules with azo groups located in the structure of MTA ligand, or by interaction with CUSs. And the increased adsorption capacity of methane can be explained by the suitable pore size of the prepared materials.

## Author contributions

Miroslav Almáši – conceptualization, data curation, formal analysis, investigation, supervision, validation, visualization, writing - original draft; writing - review & editing, Nikolas Király – data curation, formal analysis, investigation, validation, visualization, writing - original draft; writing – review & editing, Vladimír Zeleňák – funding acquisition, investigation, validation, Mária Vilková – data curation, formal analysis, investigation, validation, Sandrine Bourrelly – data curation, investigation, validation.

## Conflicts of interest

No potential conflict of interest was reported by the authors.

## Supplementary Material

RA-011-D1RA02938J-s001
