# Supplementary material for: Zinc(ii) and cadmium(ii) amorphous metal–organic frameworks (aMOFs): study of activation process and high-pressure adsorption of greenhouse gases
Source: RSC Adv. 2021 Jun 4;11(33):20137–50. doi: 10.1039/d1ra02938j (PMC9033798; doi:10.1039/d1ra02938j)
Supplement: RA-011-D1RA02938J-s001 [file RA-011-D1RA02938J-s001.pdf]

Electronic Supplementary Material (ESI) for RSC Advances.
This journal is © The Royal Society of Chemistry 2021

ELECTRONIC SUPPLEMENTARY MATERIAL

Zinc(II) and Cadmium(II) Amorphous Metal-Organic Frameworks (aMOFs): Study of Activation Process and High-pressure Adsorption of Greenhouse Gases

Miroslav Almáši[a*], Nikolas Király[a], Vladimír Zeleňák[a], Mária Vilková[b], Sandrine Bourrelly[d]

[a] Department of Inorganic Chemistry, Faculty of Science, P. J. Šafárik University, Moyzesova 11, SK-041 01 Košice, Slovak Republic

[b] NMR laboratory, Faculty of Science, P. J. Šafárik University, Moyzesova 11, SK-041 01 Košice, Slovak Republic

[c] Aix-Marseille University, CNRS, MADIREL, F-133 97 Marseille, France

[*]e-mail: miroslav.almasi@upjs.sk

Tables:

Table S1 A list of syntheses performed using different variations of synthetic conditions (molar ratio, solvents, reaction temperature and reaction time).

	Reactants	Molar ratio	Solvent (volume ratio)	Volume	Temp. / Time	Product
**1.**	Zn + H_4MTA	2:1	DMF/H_2O (6:0.5)	6.5cm^3	80°C/120hod	C
**2.**	Zn + H_4MTA	4:1	DMF/H_2O (6:0.5)	6.5cm^3	80°C/120hod	C+P
**3.**	Zn + H_4MTA	6:1	DMF/H_2O (6:0.5)	6.5cm^3	80°C/120hod	C+P
**4.**	Zn + H_4MTA	8:1	DMF/H_2O (6:0.5)	6.5cm^3	80°C/120hod	C+P
**5.**	Zn + H_4MTA	8:1	DMF/H_2O (6:0.5)	6.5cm^3	150°C/48hod	P
**6.**	Zn + H_4MTA	8:1	DMF/H_2O (6:0.5)	6.5cm^3	180°C/48hod	D
**7.**	Zn + H_4MTA	8:1	DMF/H_2O (6:0.5)	6.5cm^3	200°C/48hod	D
**8.**	Zn + H_4MTA	2:1	DMF/H_2O (6:0.5)	6.5cm^3	50°C/120hod	N
**9.**	Zn + H_4MTA	8:1	DMF/H_2O (6:0.5)	6.5cm^3	50°C/120hod	N
**10.**	Zn + H_4MTA	2:1	DMF/H_2O (6:0.5)	6.5cm^3	60°C/120hod	N
**11.**	Zn + H_4MTA	8:1	DMF/H_2O (6:0.5)	6.5cm^3	60°C/120hod	N
**12.**	Zn + H_4MTA	2:1	DMF/H_2O (6:1)	7cm^3	80°C/120hod	P
**13.**	Zn + H_4MTA	8:1	DMF/H_2O (6:1.5)	7.5cm^3	80°C/120hod	P
**14.**	Zn + H_4MTA	2:1	DMA/H_2O (6:1)	7cm^3	80°C/120hod	P
**15.**	Zn + H_4MTA	8:1	DMA/H_2O (6:2)	8cm^3	80°C/120hod	P
**16.**	Zn + H_4MTA	2:1	DMF/H_2O (6:0.5)	6.5cm^3	120°C/120hod	P
**17.**	Zn + H_4MTA	8:1	DMF/H_2O (6:0.5)	6.5cm^3	120°C/120hod	P
**18.**	Zn + H_4MTA	2:1	DMF	6.5cm^3	120°C/120hod	P
**19.**	Zn + H_4MTA	8:1	DMF	6.5cm^3	120°C/120hod	P
**20.**	Zn + H_4MTA	2:1	DMF/H_2O (6:0.5)	6.5cm^3	70°C/120hod	C
**21.**	Zn + H_4MTA	8:1	DMF/H_2O (6:0.5)	6.5cm^3	70°C/120hod	C
**22.**	Zn + H_4MTA	0.5:1	DMF/H_2O (6:0.5)	6.5cm^3	80°C/120hod	N
**23.**	Zn + H_4MTA	1:1	DMF/H_2O (6:0.5)	6.5cm^3	80°C/120hod	C+P
**24.**	Zn + H_4MTA	1.5:1	DMF/H_2O (6:0.5)	6.5cm^3	80°C/120hod	C
**25.**	Zn + H_4MTA	0.5:1	DMF/EtOH/H_2O	8.5cm^3	80°C/120hod	P

			(6:2:0.5)			
**26.**	Zn + H_4MTA	2:1	DMF/EtOH/H_2O (6:2:0.5)	8.5cm^3	80°C/120hod	P
**27.**	Zn + H_4MTA	8:1	DMF/EtOH/H_2O (6:2:0.5)	8.5cm^3	80°C/120hod	P
**28.**	Zn + H_4MTA	2:1	DMF/EtOH/H_2O (6:2:0.5)	8.5cm^3	120°C/120hod	P
**29.**	Zn + H_4MTA	8:1	DMF/EtOH/H_2O (6:2:0.5)	8.5cm^3	120°C/120hod	P
	Zn + Na_4MTA	2:1	DMF	6cm^3	80°C/120hod	P
**30.**	Zn + Na_4MTA	2:1	DMA	6cm^3	80°C/120hod	P
**31.**	Zn + Na_4MTA	2:1	H_2O	6cm^3	-	X
**32.**	Zn + Na_4MTA	2:1	EtOH/H_2O (1:1)	6cm^3	-	X
**33.**	Zn + Na_4MTA	2:1	DMF/EtOH/H_2O (6:2:0.5)	8.5cm^3	-	X
**34.**	Cd + H_4MTA	2:1	DMF/H_2O (6:0.5)	6.5cm^3	80°C/120hod	P
**35.**	Cd + H_4MTA	4:1	DMF/H_2O (6:0.5)	6.5cm^3	80°C/120hod	C+P
**36.**	Cd + H_4MTA	6:1	DMF/H_2O (6:0.5)	6.5cm^3	80°C/120hod	C+P
**37.**	Cd + H_4MTA	8:1	DMF/H_2O (6:0.5)	6.5cm^3	80°C/120hod	C
**38.**	Cd + H_4MTA	8:1	DMF/H_2O (6:0.5)	6.5cm^3	150°C/48hod	P
**39.**	Cd + H_4MTA	8:1	DMF/H_2O (6:0.5)	6.5cm^3	180°C/48hod	D
**40.**	Cd + H_4MTA	8:1	DMF/H_2O (6:0.5)	6.5cm^3	200°C/48hod	D
**41.**	Cd + H_4MTA	2:1	DMF/H_2O (6:0.5)	6.5cm^3	50°C/120hod	N
**42.**	Cd + H_4MTA	8:1	DMF/H_2O (6:0.5)	6.5cm^3	50°C/120hod	N
**43.**	Cd + H_4MTA	2:1	DMF/H_2O (6:0.5)	6.5cm^3	60°C/120hod	N
**44.**	Cd + H_4MTA	8:1	DMF/H_2O (6:0.5)	6.5cm^3	60°C/120hod	N
**45.**	Cd + H_4MTA	2:1	DMF/H_2O (6:1)	7cm^3	80°C/120hod	P
**46.**	Cd + H_4MTA	8:1	DMF/H_2O (6:1.5)	7.5cm^3	80°C/120hod	P
**47.**	Cd + H_4MTA	2:1	DMA/H_2O (6:1)	7cm^3	80°C/120hod	P
**48.**	Cd + H_4MTA	8:1	DMA/H_2O (6:2)	8cm^3	80°C/120hod	P
**49.**	Cd + H_4MTA	2:1	DMF/H_2O (6:0.5)	6.5cm^3	120°C/120hod	P
**50.**	Cd + H_4MTA	8:1	DMF/H_2O (6:0.5)	6.5cm^3	120°C/120hod	P

**51.**	Cd + H_4MTA	2:1	DMF	6.5cm^3	120°C/120hod	P
**52.**	Cd + H_4MTA	8:1	DMF	6.5cm^3	120°C/120hod	P
**53.**	Cd + H_4MTA	2:1	DMF/H_2O (6:0.5)	6.5cm^3	70°C/120hod	P
**54.**	Cd + H_4MTA	8:1	DMF/H_2O (6:0.5)	6.5cm^3	70°C/120hod	C
**55.**	Cd + H_4MTA	0.5:1	DMF/H_2O (6:0.5)	6.5cm^3	80°C/120hod	N
**56.**	Cd + H_4MTA	1:1	DMF/H_2O (6:0.5)	6.5cm^3	80°C/120hod	N
**57.**	Cd + H_4MTA	1.5:1	DMF/H_2O (6:0.5)	6.5cm^3	80°C/120hod	P
**58.**	Cd + H_4MTA	0.5:1	DMF/EtOH/H_2O (6:2:0.5)	8.5cm^3	80°C/120hod	P
**59.**	Cd + H_4MTA	2:1	DMF/EtOH/H_2O (6:2:0.5)	8.5cm^3	80°C/120hod	P
**60.**	Cd + H_4MTA	8:1	DMF/EtOH/H_2O (6:2:0.5)	8.5cm^3	80°C/120hod	P
**61.**	Cd + H_4MTA	2:1	DMF/EtOH/H_2O (6:2:0.5)	8.5cm^3	120°C/120hod	P
**62.**	Cd + H_4MTA	8:1	DMF/EtOH/H_2O (6:2:0.5)	8.5cm^3	120°C/120hod	P
**63.**	Cd + Na_4MTA	2:1	DMF	6cm^3	80°C/120hod	P
**65.**	Cd + Na_4MTA	2:1	DMA	6cm^3	80°C/120hod	P
**66.**	Cd + Na_4MTA	2:1	H_2O	6cm^3	-	X
**67.**	Cd + Na_4MTA	2:1	EtOH/H_2O (1:1)	6cm^3	-	X
	Cd + Na_4MTA	2:1	DMF/EtOH/H_2O (6:2:0.5)	8.5cm^3	-	X

Zn = $Zn(NO_3)_2 \cdot 6H_2O$, Cd = $Cd(NO_3)_2 \cdot 4H_2O$, H_4MTA = methanetetrayltetrakis(benzene-4,1-diyl)tetrakis(aza))tetrakis(methan-1-yl-1-yliden)tetrabenzoic acid, Na_4MTA = tetrasodium methanetetrayltetrakis(benzene-4,1-diyl)tetrakis(aza))tetrakis(methan-1-yl-1-yliden) tetrabenzoate, DMF = N, N´- dimethylformamide, DMA = N, N´- dimethylacetamide, EtOH = ethanol, H_2O = water, C = quasi-crystal, P = powder, N = no product, D = decomposition, X = precipitate (product precipitate after mixing of the reactant solutions)

Figures:

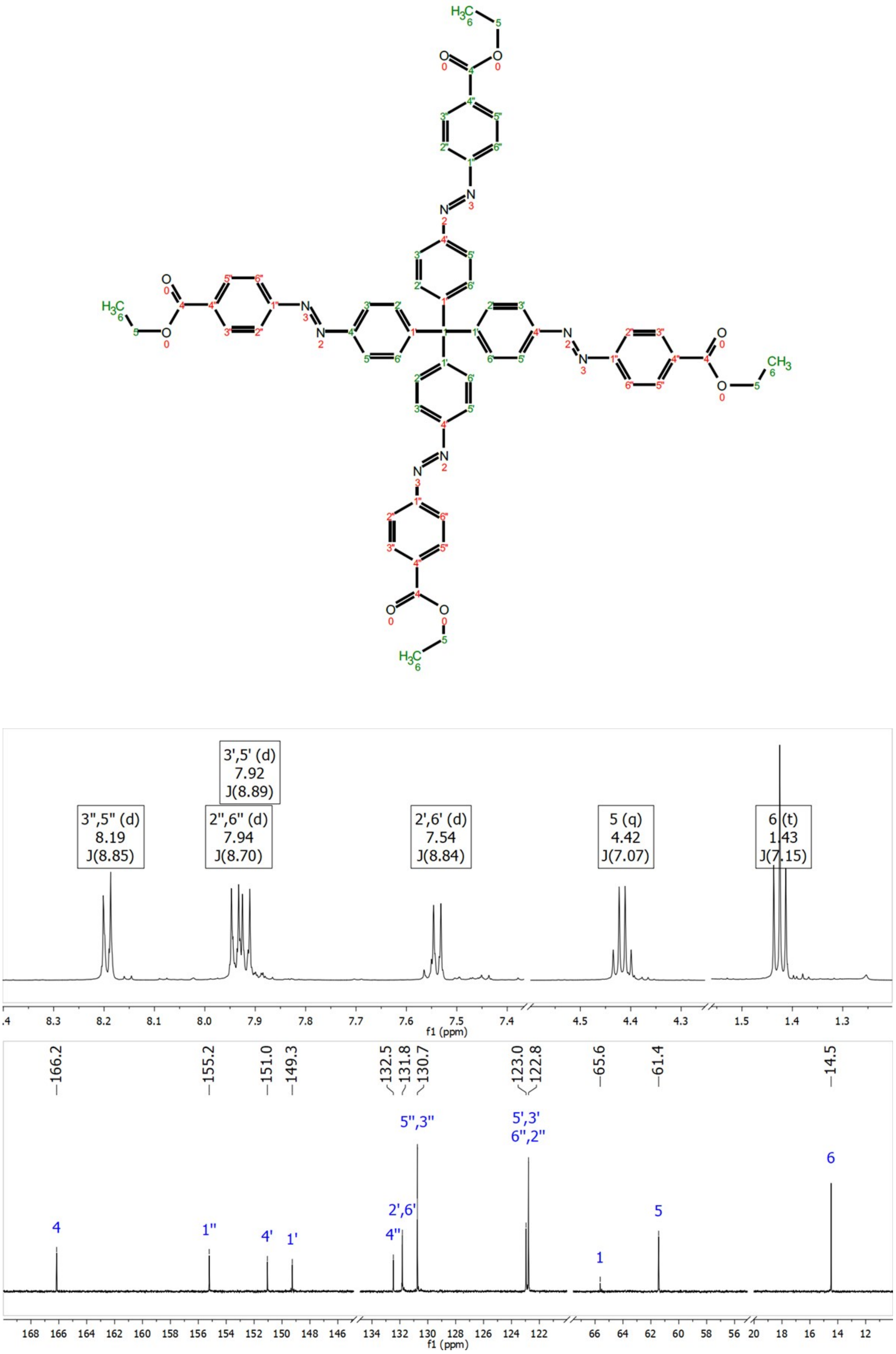

Fig. S1 ^{1}H (600 MHz, DMSO-d_6) and ^{13}C (151 MHz, DMSO-d_6) NMR spectra of the derivative **6**.

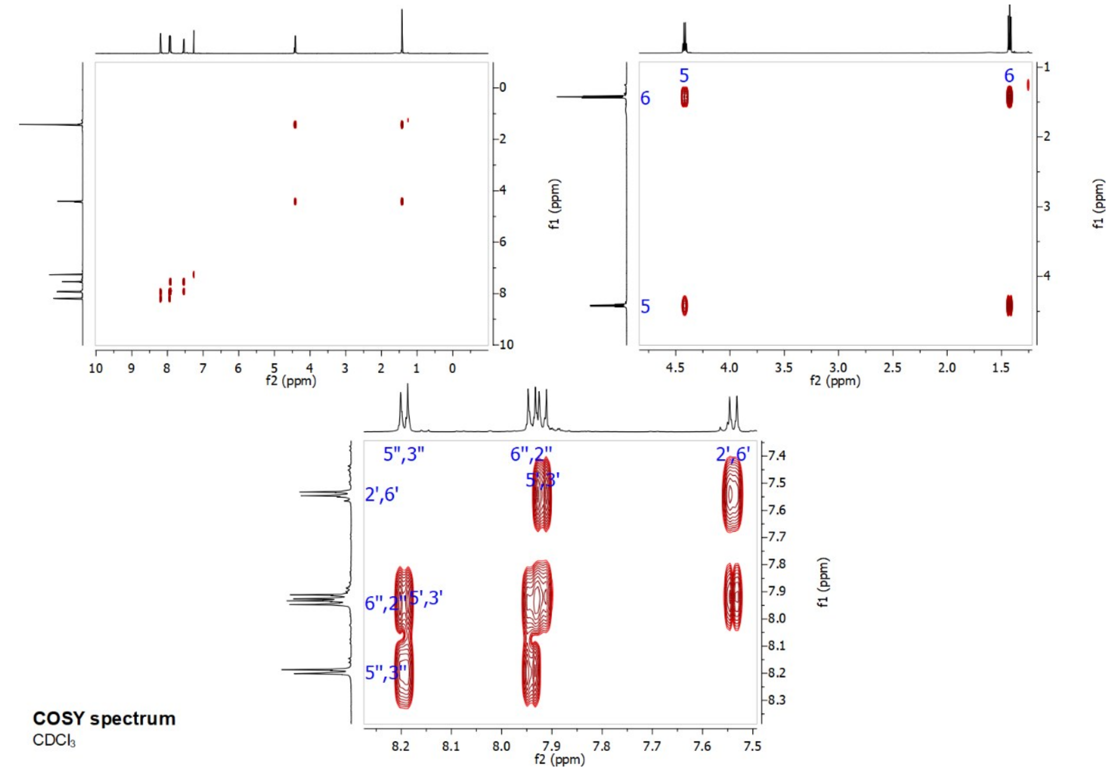

Fig. S2 ^{1}H,^{1}H-COSY spectrum (600/600 MHz, DMSO-d$_6$) of the derivative **6**.

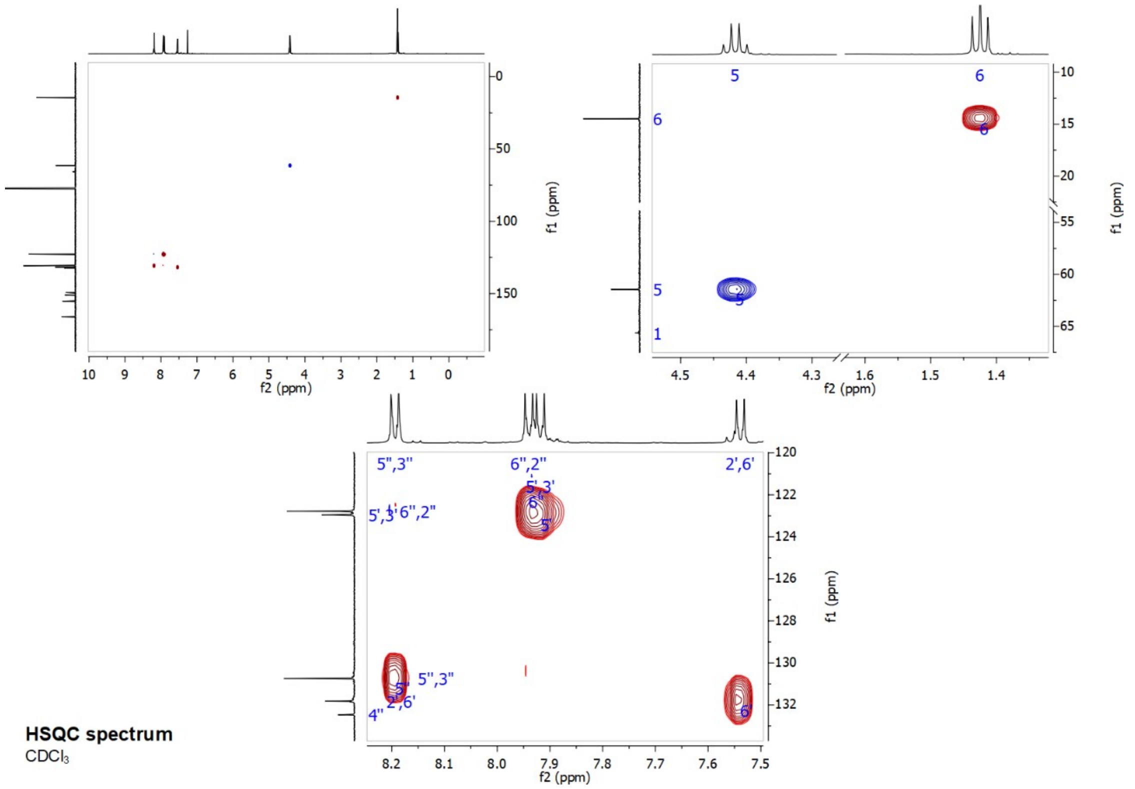

Fig. S3 ^{1}H,^{13}C-HSQC spectrum (600/151 MHz, DMSO-d$_6$) of the derivative **6**.

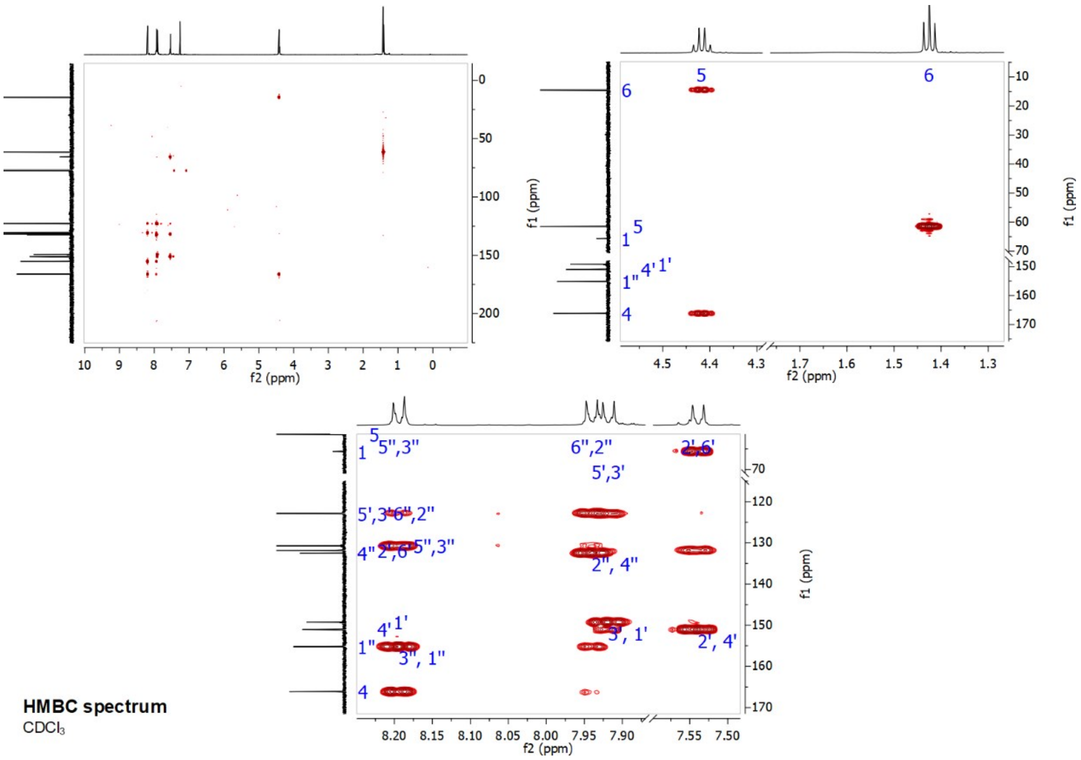

Fig. S4 ^{1}H,^{13}C-HMBC spectrum (600/151 MHz, DMSO-d$_6$) of the derivative **6**.

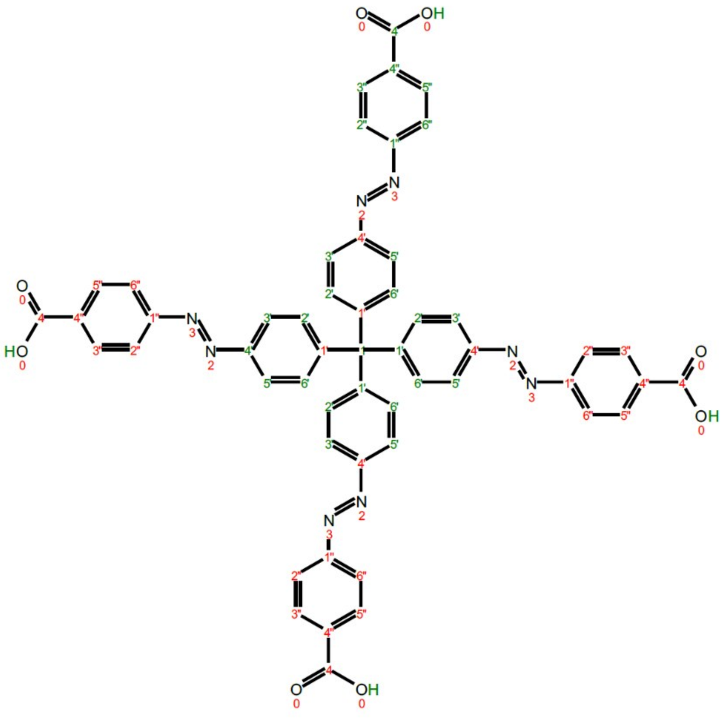

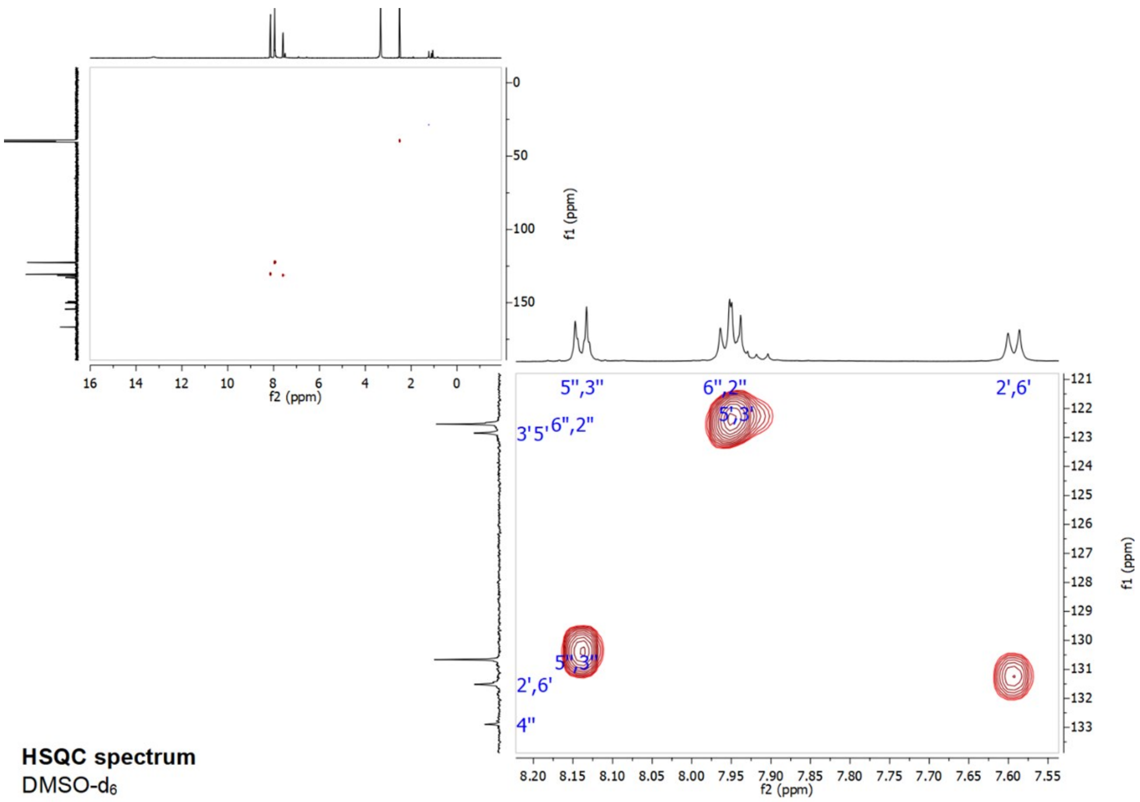

Fig. S5 $^{1}H,^{13}C$-HSQC spectrum (600/151 MHz, DMSO-d_6) of the derivative **7**.

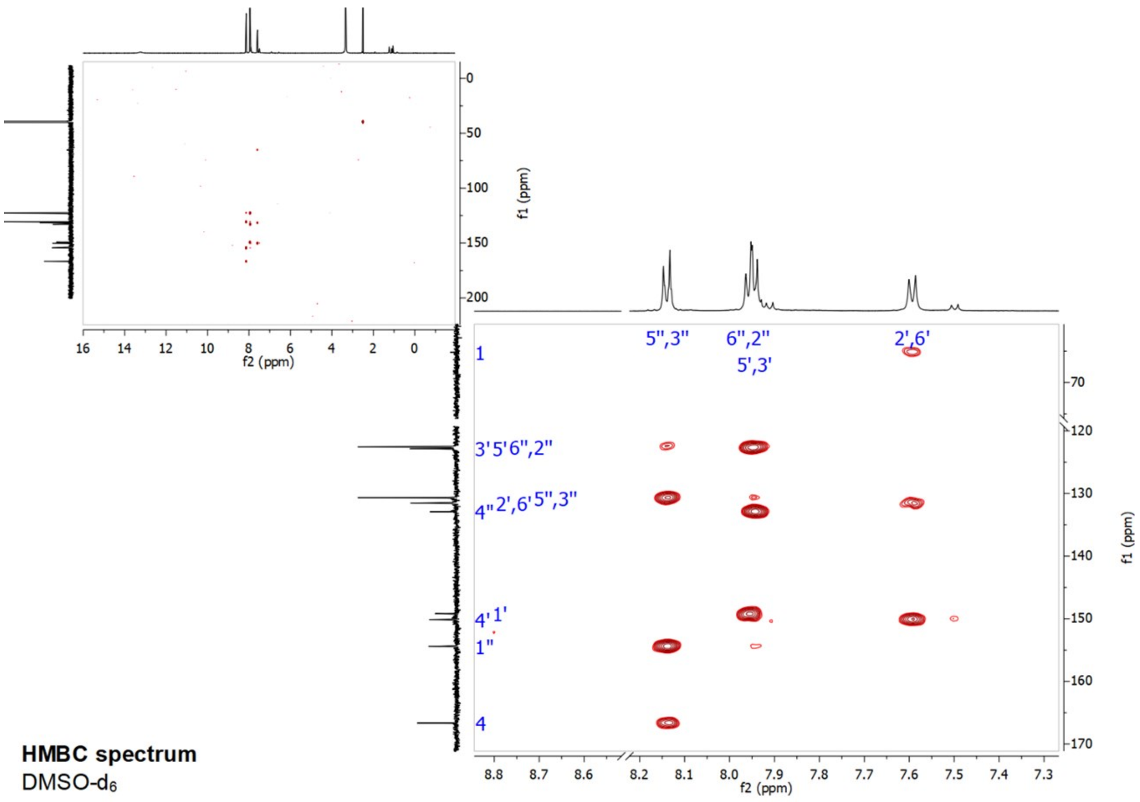

Fig. S6 $^{1}H,^{13}C$-HMBC spectrum (600/151 MHz, DMSO-d_6) of the derivative **7**.
